# Social Cognition and Obsessive-Compulsive Disorder: A Review of Subdomains of Social Functioning

**DOI:** 10.3389/fpsyt.2020.00118

**Published:** 2020-03-13

**Authors:** Myrthe Jansen, Sandy Overgaauw, Ellen R. A. De Bruijn

**Affiliations:** ^1^ Department of Clinical Psychology, Institute of Psychology, Leiden University, Leiden, Netherlands; ^2^ Leiden Institute for Brain and Cognition (LIBC), Leiden University, Leiden, Netherlands

**Keywords:** social cognition, obsessive-compulsive disorder, social cue perception, facial emotion recognition, mentalizing / theory of mind, empathy, emotion experience, emotion regulation

## Abstract

Disturbances in social cognitive processes such as the ability to infer others' mental states importantly contribute to social and functional impairments in psychiatric disorders. Yet, despite established social, emotional, and cognitive problems, the role of social cognition in obsessive-compulsive disorder is largely overlooked. The current review provides a first comprehensive overview of social (neuro)cognitive disturbances in adult patients with obsessive-compulsive disorder. Results of our review indicate various social cognitive alterations. Patients with obsessive-compulsive disorder show deficits in the recognition of affective social cues, specifically facial expressions of disgust, and more general deficits in theory of mind/mentalizing. Additionally, patients show heightened affective reactions and altered neural responding to emotions of self and others, as well as poor emotion regulation skills, which may contribute to poor social functioning of patients. However, the discrepancies in findings and scarcity of studies make it difficult to draw firm conclusions with regard to the specificity of social cognitive disturbances. The review offers directions for future research and highlights the need to investigate obsessive-compulsive disorder from an interactive social neurocognitive perspective in addition to the prevalent passive spectator perspective to advance our understanding of this intricate and burdensome disorder.

## Introduction

Essentially, almost all psychiatric disorders are characterized by disturbances in the ability to have successful and meaningful interactions with others. As such, a novel suggestion has been to reconstruct the social difficulties observed in psychiatric disorders as disorders of social cognition (1). Social cognition is a broad term that includes a wide variety of interrelated cognitive processes that enable successful and adaptive behavior in a social context [e.g., (2, 3)]. It includes, among other things, the ability to recognize social cues such as facial emotions, the ability to understand others' mental states [known as theory of mind (ToM) or mentalizing], the ability to share the experiences and emotions of others, as well as the capacity to regulate one's emotional responses to others (4). Disturbances in these social cognitive abilities are important predictors of social and functional impairments in psychiatric disorders [e.g., (5)].

Obsessive-compulsive disorder (OCD) is a burdensome psychiatric illness with a lifetime prevalence of 1%–3% (6). The disorder is characterized by the presence of unwanted, persistent obsessions that cause significant anxiety or distress, often in combination with compulsions, which are repetitive ritualistic behaviors or mental acts carried out in response to obsessions to ease distress or anxiety (7). Obsessions can range from a fear of contamination to the experience of intrusive violent or sexually explicit thoughts or images, while compulsions may include repeated checking, washing, cleaning, and counting (7). These symptoms carry a great emotional and social burden on patients as well as their relatives. Indeed, quality of life is significantly impaired in OCD patients, with social and emotional functioning being among the most greatly affected quality of life domains (8). Scores on psychosocial functioning are also lower compared to most other psychiatric disorders, and similar to schizophrenia, which is considered one of the most severe psychiatric disorders in terms of social impairments (9). Moreover, higher symptom severity has been found to be associated with poorer social adjustment (10). The extent to which these self-reported social impairments of patients with OCD simply result as a consequence of the invalidating nature of the disorder, e.g., when a patient is not able to establish or maintain meaningful relations with others because their compulsions take up too much time, or whether factors more directly related to their symptomatology such as social-cognitive problems may play a role as well, is currently unknown.

Despite these acknowledged social difficulties in OCD, research up to date has been largely limited to nonsocial cognition. This research has demonstrated that patients with OCD are characterized by meta-cognitive biases such as (moral) thought-action fusion, which is the belief that having unwanted and intrusive thoughts is (morally) equivalent to acting on these thoughts [see, e.g., (11)]. Furthermore, neuropsychological research has described that patients show cognitive deficits in a wide range of domains, including response inhibition, interference control, cognitive flexibility, and executive functioning, although findings are somewhat inconsistent [for a recent review, see (12)]. More consistently, increased performance or error monitoring has been demonstrated in OCD [for a recent review see (13)]. Given that cognitive abilities are thought to be integral aspects of social cognitive skills such as mentalizing [e.g., (14)], impairments in these abilities may also have important implications for the social cognitive functioning of patients.

Neuroimaging studies in patients with OCD suggest that dysfunctions in cortical-striatal-thalamic-cortical circuitry underlie aforementioned cognitive deficits [e.g., (15)]. More recent work specifically implicates the lateral and medial orbitofrontal cortices, (dorsal) anterior cingulate cortex (ACC), and amygdala-cortical circuitry in the psychopathology of the disorder (16, 17). The insular cortex, a brain area involved in, among other things, the processing of disgust (18), is also implicated in the disorder. Hyperactivity of this region is commonly reported during symptom provocation, especially in those with contamination-related obsessions (19–21). The performance monitoring account of OCD also proposes a central role for both the ACC and the insular cortex. This account suggests that these brain areas are involved in producing persistent high error or conflict signals which patients are unable to reduce by behavioral action, resulting in repeated actions (i.e., compulsions) in an attempt to temper such signals (22). This theory is supported by findings of enhanced amplitudes of an event-related potential (ERP) component related to error detection called the error-related negativity [ERN; (23, 24)] in patients with OCD [see (13)]. This component is thought to be generated in the ACC (25), thus highlighting the importance of this area in the psychopathology of the disorder.

Importantly, many of the brain areas known to be implicated in the psychopathology of OCD, such as the amygdala, ACC, and insula, are also areas known to be involved in social cognitive processes and are considered to be part of the social brain in general (26–29). ToM abilities for example, have been shown to involve a network of brain regions also implicated in OCD including the amygdala, ACC, as well as other prefrontal regions (29). The amygdala and insula are both implicated in the perception of facial expressions of emotions as well (28). Furthermore, social influences have been shown to importantly modulate electrophysiological measures and brain regions involved in cognitive processes such as performance monitoring [for a review see (30)]. Yet, while research shows that many cognitive functions and brain areas involved in social behavior and cognition are affected in patients with OCD, research has largely overlooked the implications of these anomalies for social cognitive functioning and associated symptomatology in this disorder.

Identifying social cognitive disturbances has great functional relevance, as this may advance our understanding of altered social functioning of patients with OCD and lead to an improved characterization of the phenotype of this disorder. It may also have important therapeutic implications, as recent studies are increasingly starting to recognize the potential of social cognition as a target for clinical intervention [see, e.g., (31–33)]. A previous meta-analysis focusing on various anxiety disorders showed social cognitive deficits with small to moderate effect sizes for patients with OCD (34). This however concerned an exclusively quantitative analysis covering a limited number of studies (N=14, of which 12 concerned facial emotion recognition). As a result, to this date, social cognition in OCD is still poorly understood. The current review therefore aims to advance our understanding of social cognition in this disorder by qualitatively reviewing existing studies on this topic. As there are many different perspectives on what processes or domains can be considered as social cognition, we decided to adopt the framework used by Green et al. (4) in their widely cited review paper on social cognition in schizophrenia. The authors of this paper divided subdomains of social cognition according to “recent organizational models of neural systems in social neuroscience” (4 p. 620). We will therefore focus on these same domains: “social cue perception,” “mentalizing/ToM,” “experience sharing and empathy,” and “emotion experience and regulation.”

## Social Cue Perception

The way people act, move, speak, gesture, and express their emotions conveys important social information. How we perceive, identify or interpret these social cues expressed by other people essentially determines how we interact with others. The following section will focus on how patients with OCD perceive affective (*Affective Social Cues*) as well as nonaffective social cues (*Nonaffective Social Cues*). **Table 1** contains an overview of the studies discussed in this section.

**Table 1:** Overview of studies investigating the perception of social cues in obsessive-compulsive disorder.

Domain	Author	Method	Participants	Comorbid diagnoses?	Concurrent medication/therapy?	Task description	Subdomain	Emotions assessed	Diagnosis/symptom assessment	Main results
*Affective cues*										
	Aigner et al. (35)	Case-control Outpatients	OCD = 40 [34.8 ± 10.4, 24M:16F]; HC = 40 [34.7 ± 8.7, 24M:16F]	None.	All patients were treated with SSRIs. Therapy not reported.	Static tasks (EMODIFF (differentiate emotions) and PEAT (rate valence from very sad – very happy)	Facial emotion recognition	Happiness, sadness, neutral	DSM-IV, SCID/Y-BOCS	OCD patients less accurate in identifying sad faces on the PEAT, but only of females (p=0.034). They also showed a bias to recognize neutral as sad (p=0.029), happy faces as neutral (p=0.022) and happy as sad (p=0.024).
	Bozikas et al. (36)	Case-control Outpatients	OCD = 25 [32.7 ± 9.0, 10M:15F]; HC = 25 [33.4 ± 7.3, 14M:11F]	Depression (n=4), PD (n=2).	Antidepressants without (n=11) and with (n=5) atypical antipsychotics, antipsychotics only (n=1). All patients were receiving CBT.	KAMT; Static matching task	Facial emotion recognition + affective prosody	Happiness, surprise, sadness, anger, fear, disgust	DSM-IV, MINI (4.4)/Y-BOCS	Compulsion subscale correlated significantly with sadness recognition (p=0.006). Total Y-BOCS scores correlated significantly with fear recognition (p=0.042). Associations did not survive Bonferroni correction.
	Buhlmann et al. (37)	Case-control Outpatients	OCD = 20 [31.0 ± 10.5, 8M:12F]; HC = 20 [32.9 ± 11.7, 7M:13F]	Not reported.	Not reported.	Static labelling task	Facial emotion recognition	Happiness, surprise, sadness, anger, fear, disgust, neutral	SCID	No differences between OCD and HC.
	Cannistraro et al. (38)	Case-control fMRI	OCD = 10 [26.8 ± 5.2, 4M:6F]; HC = 10 [24.9 ± 7.8, 4M:6F]	One subject with comorbid GAD and BDD.	Sertraline (n=1).	Passive viewing task	Facial emotion recognition	Happiness, fear, neutral	SCID/Y-BOCS	Compared to HC, OCD patients exhibited attenuated activation in both left (p=0.008) and right amygdala (p=0.023) when contrasting all facial expressions with fixations.
	Cardoner et al. (39)	Case-control Outpatients fMRI	OCD = 21 [28.52 ± 5.9, 10M: 11F]; HC = 21 [26.2 ± 3.4, 10M: 11F]	Depression and ADs (n=7), MDD (n=2), GAD (n=2), SAD (n=2), PD (n=1).	Fluoxetine (n=4), fluvoxamine (n=2), citalopram (n=1), clomipramine (n=2), clomipramine with SSRI (n=11). Therapy not reported.	Emotional Face Matching Task: static matching task	Facial emotion recognition	Happiness, fear (and anger)	DSM-IV/Y-BOCS	OCD patients were less accurate in matching both emotional faces as well as nonemotional shapes compared to HC (p=0.04). OCD patients showed significantly enhanced activation of visual striate areas, right fusiform gyrus, left posterior thalamus, right amygdala and parahippocampal cortex as well as dorsolateral prefrontal and right premotor cortex when comparing trials with emotional faces versus nonemotional shapes (p < 0.005, whole–brain uncorrected).
	Corcoran et al. (40)	Case-control	OCD = 40; HC = 36. Overall mean age was 34.0 years ± 11.1), no group differences in age or gender (63% women).	Comorbidities: MDD (32.5%), AD (13.9%).	Not reported.	Hexagon labelling task	Facial emotion recognition	Sadness, anger, fear, disgust	ADIS-IV or SCID/Y-BOCS	OCD patients were significantly less accurate than HC in recognizing disgust (p < 0.01). Within the OCD group, 27 individuals were unimpaired in disgust recognition (MHR = 22.6, SD = 1.7), while 13 individuals were impaired (MHR = 12.9, SD = 3.4). Patients with impairments scored significantly higher on the Y-BOCS and lower on the GAF.
	Daros et al. (41)	Meta-analysis Case-control	OCD = 221 [30.4 ± 7.6, 102M:119F]; HC = 224 [30.9 ± 8.8, 102M: 122F]	Not reported in all studies included in the meta-analysis. Approximately 30% has at least one comorbid AD and approximately 13% had comorbid MDD.	Not reported in all studies included in the meta-analysis. Based on information from 3 studies, approximately 59% taking psychotropic medication, most commonly antidepressants.	Only studies using labelling tests were included. Studies using blended emotions were included only if they included stimuli at 100% intensity.	Facial emotion recognition	Happiness, surprise, sadness, anger, fear, disgust, neutral	DSM-III-R or DSM-IV diagnosed	Overall emotion recognition accuracy was lower in patients compared to controls (d=−0.55; N=11; 95% CI= −0.92 to −0.19,p=0.03) with larger effects for static (d=−0.77, N=7, 95% CI= −1.23 to −0.32, p=0.01) compared to morphed expressions (d=−0.14, N=4, 95% CI=−0.51 to 0.24, p=0.48). Recognition of overall negative emotions was also impaired l (d=−0.34, N=11, 95% CI=−0.56 to −0.11, p < 0.01), as were disgust (d=−0.59, N=11, 95% CI= −1.06 to −0.11, p=0.02), anger (d=−0.36, N=10, 95% CI=−0.67 to −0.05, p=0.02) and sadness (d=−0.31, N=10, 95% CI=−0.062 to 0.00, p=0.05) separately.
	Jhung et al. (42)	Case-control Outpatients	OCD = 41 [24.9 ± 5.3, 32M: 9F]; HC = 37 [26.0 ± 6.0, 28M: 9F]	Comorbid diagnoses were allowed but no specifics are provided.	Not reported.	Hexagon labelling task (incl. ambiguous faces)	Facial emotion recognition	Sadness, anger, fear, disgust	SCID/Y-BOCS	After adjusting for age, sex and depression, patients were significantly more likely to perceive ambiguous emotions as disgust (p=0.005) and less likely to perceive them as anger (p=0.008). Higher cleaning scores predicted lower perception of anger (p=0.01) and greater perception of disgust in ambiguous expressions before (p=0.003) and after controlling for covariates (p=0.005). Hoarding predicted poorer recognition of nonambiguous disgust before (p=0.049) but not after controlling for covariates (p=0.08).
	Kornreich et al. (43)	Case-control Outpatients	OC = 22 [37.3 ± 8.0, M/F =9/13]; NC = 22 [37.2 ± 9.0, M/F = 9/13]	Not reported.	All OCD patients were being treated with SSRIs. Therapy not reported.	Labelling task with morphed expressions (30 or 70% neutral)	Facial emotion recognition	Happiness, surprise, sadness, anger, fear, disgust, shame, contempt	DSM-IV/Y-BOCS	No significant differences between patients and HC for accuracy nor intensity.
	Lawrence et al. (44)	Case-control 7 inpatients, 10 outpatients fMRI	OCD = 17 [34.9 ± 8.2, 10M: 7F]; HC = 19 [34.0 ± 9.4, 11M: 8F]	MDD (n=1 present, n=3 past), DD (n= 4), SP (n=1), PD (n=1), PDA (n=1), PTSS (n=1), BDD (n=1). Personality disorders: avoidant (n=5), obsessive-compulsive (n=3), depressive (n=1), paranoid (n=1), and borderline (n=1).	Citalopram (n=2), clomipramine (n=1), fluoxetine (n=3), fluvoxamine (n= 1), paroxetine (n= 4), venlafaxine (n=1), zopiclone (n=1) and buspirone (n=1). Therapy not reported.	Static labelling task (behavior) + backward masking paradigm (fMRI)	Facial emotion recognition	Fear, disgust, neutral	SCID/Y-BOCS	No behavioral differences between OCD patients and HC nor between high- or low washing patients. Compared to HC, OCD patients showed enhanced activation in the left ventrolateral prefrontal cortex and reduced activation in the thalamus when contrasting facial expressions of disgust with neutral expressions (ps < 0.05). This pattern was especially pronounced for patients with more washing symptoms.
	Lochner et al. (45)	Randomized double-blind case-controlled crossover study	OCD = 20; [34.1 ± 11.0, 11M: 9F]; subgroups: OCD with SSRI treatment (n =11); OCD without SSRI treatment (n=9); HC = 20 [34.8 ± 10.8, 9M: 11F]	Specific phobia (n = 2).	Sertraline (n=3), fluoxetine (n=5), escitalopram (n=1), citalopram (n=1) and paroxetine (n=1). Therapy not reported.	Labelling task with morphing video clips modified from Montagne et al. (46)	Facial emotion recognition	Happiness, sadness, anger, fear, disgust	MINI-plus, ICD-10/Y-BOCS	OCD severity was marginally associated with decreased disgust recognition after adjusting for Y-BOCS and MADRS (p=0.06). On placebo, accuracy was similar across groups. OCD patients on SSRIs showed significantly increased disgust recognition after escitalopram challenge compared to when they were on placebo and compared to the other two groups.
	Mavrogiorgou et al. (47)	Case-control Outpatients	OCD = 20 [38.1 ± 10.6, 12M: 8F]; HC = 20 [38.2 ± 13.0, 12M: 8F]	Comorbid MDD and ADs not considered as exclusion criteria.	SSRIs (n=18), St. John's wort (n=1), SSRIs + antipsychotic drugs (n=13). CBT treatment were not considered as exclusion criteria.	Static labelling task.	Facial emotion recognition	Happiness, surprise, sadness, anger, fear, disgust	ICD-10, DSM-IV criteria/Y-BOCS	No significant difference between patients and HC in emotion recognition (p > 0.5).
	Montagne et al. (46)	Case-control	OCD = 21 (9M: 12F; Subgroups: HRAC [n=13; 36.6 ± 11.3, 5M:8F], CC [n= 5; 41.6 ± 9.1, 3M:2F] and PS [n=3; 24.3 ± 4.5, 1M:2F]; HC = 47 [40.6 ± 12.3, 24M:23F]	Not reported.	All patients were medication-free for at least 4 weeks prior to testing. Therapy not reported.	Labelling task with morphing video clips	Facial emotion recognition	Happiness, surprise, sadness, fear, disgust	MINI for DSM-IV/Y-BOCS	Patients from HRAC group needed less emotional intensity than HC to recognize facial expression of fear (p < 0.02) and happiness (p < 0.04) correctly.
	Parker et al. (48)	Case-control Outpatients	OCD = 15 [37.7 ± 10.2, 7M:8F]; HC = 15 [31.3 ± 12.2, 3M: 12F]	Not reported.	Most were receiving behavioral and/or psychopharmacologic treatment.	Hexagon labelling task	Facial emotion recognition	Happiness, surprise, sadness, anger, fear, disgust	DSM-IV criteria/Y-BOCS	For disgust recognition, there was no significant difference between OCD patients and HC, although one OCD subject showed markedly poor disgust recognition.
	Rector et al. (49)	Case-controlled cross-sectional study Tertiary care clinic	OCD without CBT treatment = 20; PDA = 15; GSP= 10; OCD + treatment responders to CBT = 11. Overall age was 33.6 ± 8.5 years), 55% women. Characteristics per group not reported.	Exclusion criteria included concurrent diagnosis of a mood disorder, SSDs, PDA or GSP.	Patients were on stable medication (no change in medication type or dose during 8 weeks prior to testing). No specifics reported. Exclusion criteria included past treatment with CBT.	Static labelling task	Facial emotion recognition	Happiness, surprise, sadness, anger, fear, disgust	SCID/Y-BOCS	Untreated OCD group performed significantly worse on disgust recognition than the PDA (p < 0.05), GSP (p < 0.01) and OCD group treated with CBT (p < 0.05). OCD group treated with CBT recognized anger significantly better than untreated OCD (p < 0.05) and PDA group (p < 0.05).
	Sprengelmeyer et al. (50)	Case-control	OCD = 12 [34.8 ± 10.1, 5M:7F]; HC (task 2) = 40 [42.9 ± 14.3, 19M:21F]	Not reported.	Not reported.	Both a hexagon and a static labelling task	Facial emotion recognition	Happiness, surprise, sadness, anger, fear, disgust	DSM-III-R	OCD group significantly less accurate in recognizing disgust in the emotional hexagon task than HC (p < 0.001) and somewhat less accurate in recognizing anger (0.01 > p < 0.05). Recognition of static expressions of disgust was also significantly impaired in the OCD group (p < 0.01).
	Toh et al. (51)	Case-control Outpatients	OCD = 19 [37.0 ± 10.4, 5M: 14F]; BDD = 21 [34.3 ± 11.9, 5M: 16F]; HC = 21 [35.7 ± 10.6, 8M: 13F]	SAD (n=2), MDD (n=6).	SSRIs (n=10), SNRIs (n=3), TCAs (n = 1), with some receiving atypical antipsychotic augmentation (n = 4). Therapy not reported.	Static labelling task.	Facial emotion recognition	Happiness, surprise, sadness, anger, fear, disgust, neutral	MINI500/Y-BOCS	BDD group were less accurate overall compared to the OCD and HC groups (comparison OCD and HC not mentioned). Severe OCD was associated with poorer emotion recognition.
	Via et al. (52)	Case-control fMRI	OCD = 67 [33.1 ± 8.5, 38M:29F]; HC = 67 [32.8 ± 10.2, 38M:29F]	MDD (n=4), DD (n=2), GAD (n=3), PD (n=4), SP (n=3).	Citalopram (n=2), clomipramine (n=29), clomipramine + SSRI (n=9), escitalopram (n=7), fluoxetine (n=7), fluoxetine + SSRI (n=1), fluvoxamine (n=4), fluvaoxamine + SSRI (n=3),phenelzine (n=1), sertraline (n=1), sertraline + SSRI (n=1), adjunct antipsychotica (n=12)	Modified Emotional Face Matching Task (static matching task)	Facial emotion recognition	Happiness, fear (and anger)	SCID/DY-BOCS	No behavioral differences between patients and HC in matching of emotional faces. Compared to HC, patients exhibited enhanced activation in bilateral amygdala, and secondary visual cortex extended to intraparietal sulcus, right anterior insula cortex, premotor cortex, right orbitofrontal cortex and right middle temporal gyrus (ps < 0.05) when matching fearful faces compared to matching shapes. Only left amygdala survived whole brain level correction.
Domain	Author	Method	Participants	Comorbid diagnoses?	Concurrent medication/therapy?	Task description	Subdomain	Emotions assessed	Diagnosis/symptom assessment	Main results
***Nonaffective cues***										
	Jung et al. (53)	Case-control fMRI	OCD = 15 [23.4 ± 4.7, 12M:3F]; HC = 15 [25.67 ± 3.46, 9M:6F]	Comorbid axis I diagnoses were considered exclusion criteria.	Monoamine oxidase inhibitors (n=2), SSRI + antianxiety (n=3), SSRI + antianxiety + anti-psychotics (n=3). Therapy not reported.	One-back task with biological and scrambled motion	Biological motion perception	Not applicable.	SCID for DSM-II/Y-BOCS	Compared to HC, patients exhibited increased activation in the right superior and middle temporal gyrus, the left inferior temporal and fusiform gyrus, and reduced activation in the right postcentral gyrus (p < 0.001, uncorrected).
	Kim et al. (54)	Case-control Outpatients	OCD = 20 [24.3 ± 6.2, 12M:8F]; HC = 16 [23.2 ± 5.8, 11M:5F]	Not reported.	Sertraline (n=4), citalopram (n=6), fluoxetine (n=5), fluvoxamine (n=2), risperidone (n=5), olanzapine (n=1), clonazepam (n=14), valproic acid (n=1), and lamotrigine (n=1). Therapy not reported.	Biological motion detection and discrimination tasks	Biological motion perception	Not applicable.	DSM-IV criteria/Y-BOCS	Patients found it more difficult to detect biological motion within noise dots (p=0.003) and to discriminate biological motion from scrambled motion (p=0.034), whereas their ability to perceive nonbiological global motion and static global form was comparable to HC.
	Shin et al. (55)	Case-control Outpatients	OCD = 54 [25.0 ± 6.5, 32M:22F]; HC = 42 [23.4 ± 4.6, 32M:10F]	Comorbid axis I diagnoses were considered exclusion criteria.	Medication-naïve (n=24), medication-free for 4 weeks (n=30). Therapy not reported.	Body and face discrimination task	Recognition of faces and bodies	Not applicable.	SCID/	Compared to HC, patients were less accurate in discriminating human bodily postures (p < 0.001), but not in discriminating faces or chairs.

OCD, obsessive-compulsive disorder; HC, healthy controls; SSRIs, selective serotonin reuptake inhibitors; EMODIFF, The Facial Emotion Intensity Differentiation Test; PEAT, Penn’s Emotion Acuity Test; DSM, Diagnostic and Statistical Manual of Mental Disorders; SCID, Structured Clinical Interview for DSM Axis I Disorders; Y-BOCS, Yale–Brown Obsessive-Compulsive Scale; OAD, other anxiety disorders; AD, anxiety disorder; ODD, oppositional defiant disorder; SCL-90, Symptom Checklist-90; PD, panic disorder; GAD, generalized anxiety disorder; MDD, major depression disorder; ADHD, attention-deficit hyperactivity disorder; TCA, tricyclic antidepressants; KSADS-PL, Kiddie Schedule for Affective Disorders and Schizophrenia; YGTSS, the Yale Global Tic Severity Scale; CBT, cognitive-behavioral therapy; KAMT, Kinney’s Affect Matching Test; MINI, Mini International Neuropsychiatric Interview; SAD, Social anxiety disorder; ADIS-IV, Anxiety Disorders Interview Schedule for DSM-IV; GAF, Global Assessment of Functioning; OR, Odds ratio; OCI-R, obsessive-compulsive inventory revised; MHR, mean hit rate; DD, dysthymic disorder; PDA, panic disorder with agoraphobia; SSDs, Schizophrenia spectrum disorders; PTSS, posttraumatic stress disorder; BDD, body dysmorphic disorder; ICD, International Statistical Classification of Diseases; MADRS, Montgomery–Asberg Depression Rating Scale; HRAC, High Risk Assessment and Checking; CC, contamination and cleaning; PS, Perfectionism and Symmetry; GSP, generalized social phobia; GTS, Gilles de la Tourette’s syndrome; OBS, obsessive-compulsive symptoms; SNRIs, serotonin–norepinephrine reuptake inhibitors; SP, social phobia; DY-BOCS, Dimensional Yale-Brown Obsessive-Compulsive Scale; SGA, second-generation antipsychotic; MOCI, Maudsley Obsessive Compulsive Scale; HDRS, Hamilton Depression Rating Scale.

### Affective Social Cues

Studies on how patients with OCD process affective social cues have mainly focused on our ability to identify the affective states of others from facial cues, which is generally referred to as facial emotion recognition. Other cues, such as emotion expressed in voice or body language, have received less attention. The current section will discuss research on the recognition of facial emotions (*Facial Emotion Recognition*) in adult patients (*Facial Emotion Recognition in Patients With OCD*), studies on the role of symptom severity (*The Role of Symptom Severity in Facial Emotion Recognition*), and subtype (*The Role of Symptom Subtype in Facial Emotion Recognition*), facial emotion perception biases (*Biases in Facial Emotion Recognition*) as well as on how adults with OCD process facial emotions on a neural level (*Neural Correlates of Facial Emotion Processing*). Only one study investigating nonfacial affective cues was identified, which will be discussed in the section *Affective Prosody*.

#### Facial Emotion Recognition

Studies assessing facial emotion recognition have typically assessed the recognition of what are believed to be the six basic emotions, i.e., anger, fear, sadness, disgust, happiness, and surprise. Most emotion recognition studies in patients with OCD originated from an interest in the emotional expression of disgust. Many patients are characterized by a fear of contamination, which is associated with behavioral compulsions such as washing and cleaning. Because facial expressions of disgust convey potential contamination, this emotional expression is thought to be particularly relevant to the symptomatology of OCD (56). The expression of fear seems relevant to OCD as well, since patients with OCD are characterized by high levels of anxiety, and previous studies have among others demonstrated that anxious individuals show increased attentional bias to fear- or threat-related stimuli [see (57)] including facial expressions of fear [e.g., (58)].

##### Facial Emotion Recognition in Patients With OCD

The first investigation of facial emotion recognition in patients with OCD was conducted by Sprengelmeyer and colleagues (50), over 20 years ago. Despite their small sample (12 patients), this study reported striking deficits in the recognition of the facial expression of disgust in two tests: an emotional hexagon and static test. Both tests asked patients to label the facial emotional expressions portrayed, but while one test focused on static expressions (e.g., 100% disgust), the other test using emotional hexagons, in which distinct emotional expressions were morphed (e.g., 70% disgust and 30% anger). Patients with OCD showed specific deficits in the recognition of disgust compared to healthy controls. The emotional hexagon test also indicated a marginal deficit in the recognition of anger in the patient group but not for any other emotional expressions. Parker, McNally, Nakayama, and Wilhelm (48) attempted to replicate the findings by Sprengelmeyer et al. (50) using the same tasks in a marginally larger sample (15 patients), yet failed to find any facial emotion recognition deficits in patients. In contrast, a later study in 40 patients conducted by Corcoran, Woody and Tolin (40) followed a similar procedure as the two aforementioned studies and found that overall, patients showed a specific deficit in the recognition of static expressions of disgust, but not in any other emotion.

Other studies investigated the identification of static (37, 47) or morphed emotional facial expressions (36, 42, 43) using similar tasks, yet did not reveal any significant differences between patients and healthy controls. Lawrence et al. (44) specifically investigated fear and disgust recognition, but did not observe differences in accuracy between patients and controls, despite observing differences in neural responsiveness to facial expressions of disgust (see below in *Neural Correlates of Facial Emotion Processing*). Cardoner et al. (39) and Via et al. (52) both used an active matching task in which happy and fearful target faces had to be matched with happy, fearful or angry probe faces. Although Cardoner et al. found a main group effect, showing that patients suffering from OCD were less accurate in matching both emotional faces as well as nonemotional shapes, a similar study by Via et al. found no behavioral differences between groups, in the presence of neural differences (see below in *Neural Correlates of Facial Emotion Processing*).

Two studies specifically investigated the effect of treatment on facial emotion recognition, which suggest that medication or therapy may improve or remediate disgust recognition. Lochner et al. (45) administered a single dose of the selective serotonin reuptake inhibitor (SSRI) escitalopram to OCD patients, which is an antidepressant considered as a first-line option in the treatment of OCD (59). Compared to controls, patients showed no significant deficits in the recognition of disgust in the placebo condition, although patients were significantly more accurate after a single administration of escitalopram, especially when they were already receiving SSRI treatment. Rector, Daros, Bradbury, and Richter (49) compared patients receiving cognitive-behavioral therapy (CBT) with patients not receiving CBT. Results showed that patients not receiving CBT showed significant disgust recognition deficits, whereas patients receiving therapy showed disgust recognition scores comparable to a normative sample and also showed significantly higher accuracy of anger compared to the untreated patient group.

In an attempt to clarify inconsistencies between studies, Daros, Zakzanis, and Rector (41) conducted a meta-analytic review on facial emotion recognition including ten studies in adolescent (60) and adult OCD patients (36, 37, 40, 42, 45, 48–50, 61) (not discussed in the current review as the article was not available in English)]. Based on a combined sample of 221 patients and 223 controls, the review concluded that OCD patients were significantly less accurate in identifying the six basic emotions overall compared with controls, showing a medium effect size (Cohen's *d* = −0.55), with larger effects for static (Cohen's *d* = −0.77) compared to morphed emotional expressions (Cohen's *d* = −0.14). OCD patients were also impaired in the recognition of negative emotions as a whole (Cohen's *d* = −0.34) and had particularly difficulties with the recognition of disgust (Cohen's *d* = −0.59) and anger (Cohen's *d* = −0.36). A marginally significant deficit in the recognition of sadness was also found (Cohen's *d* = −0.31), while fear recognition was not significantly impaired (Cohen's *d* = −0.09). Thus, based on these ten patients studies, OCD is associated with pronounced impairments in the recognition of facial expressions of disgust, while modest impairments in the recognition of other negative emotions, specifically anger and sadness, but not fear, are also observed.

##### The Role of Symptom Severity in Facial Emotion Recognition

Several studies additionally report on the relation between facial emotion recognition and symptom severity of patients. Although obtaining no significant emotion recognition deficits, Parker et al. (48) did show that the patient with the most severe symptoms as measured by the Yale-Brown Obsessive-Compulsive Scale [Y-BOCS; (62)] showed marked impairments in the recognition of disgust, and suggested that such impairments might only arise for severe cases. In the study by Corcoran et al. (40), most of the patients were as accurate in recognizing disgust as healthy controls. However, approximately one-third of the patient group showed marked impairments, which led to a significant overall difference between patients and controls. The authors found that those patients who were impaired on disgust recognition had higher Y-BOCS scores as well as significantly lower scores on a scale of global functioning. Lochner and colleagues (45) also report a marginally significant negative relation between symptom severity (Y-BOCS total) and disgust recognition accuracy in a morphing task after correcting for depression scores. Furthermore, a significantly negative correlation between total Y-BOCS scores and the recognition of fear was found in an emotional matching task by Bozikas and colleagues (36), but this effect did not survive Bonferroni correction. No correlation with any of the other emotions was obtained. A study by Toh, Castle, and Rossell (51) reports a negative correlation between symptom severity (Y-BOCS total) and overall facial affect recognition but do not provide any specifics since the focus of their study concerned patients with body dysmorphic disorder, for which patients with OCD served as a reference group. Other studies however, did not observe significant relations with symptom severity (47, 49, 52) and the review by Daros and colleagues (41) also was not able to detect a significant relation between symptom severity and overall emotion recognition, nor with anger or disgust individually, based on the ten studies included in their meta-analysis. Hence, overall, there does not seem to be very strong evidence for a relation between symptom severity and facial recognition impairments.

##### The Role of Symptom Subtype in Facial Emotion Recognition

So far, studies investigating the role of symptom subtype do not seem to provide clear differences in emotion recognition between different subdomains of OCD. One study specifically compared different subdomains of OCD (46). Patients were divided into three subgroups; high risk assessment and checking, contamination and cleaning, and perfectionism and symmetry. While no significant findings emerged for disgust, the study showed a significant difference between patients scoring high on risk assessment and checking and controls in sensitivity to fear and happiness, indicating that they were able to correctly identify these emotions at a lower intensity level than controls. Jhung et al. (42) showed that having more hoarding symptoms was associated with poorer disgust recognition, yet this relation did not remain after controlling for age, sex, and depression scores. Additionally, the studies by Corcoran et al. (40) and Rector et al. (49) showed no differences in disgust recognition between patients with and without primary contamination concerns.

##### Biases in Facial Emotion Recognition

Some studies have additionally demonstrated that OCS is associated with specific biases in facial emotion perception. Aigner et al. (35) used a task that required OCD patients to rate faces as neutral, happy or sad, and the degree of intensity of these emotions. Results showed that OCD patients displayed a bias to recognize neutral faces as sad, as well as a bias to recognize happy faces as neutral and happy faces as sad (35). Patients were also less accurate in identifying sad expressions, but only for female faces. One study also indicates that patients with OCD may have bias toward perceiving faces as disgusting (42). This study investigated how patients responded to ambiguous faces (e.g., 50% disgust and 50% anger). They found that, compared to controls, OCD patients were significantly more likely to perceive ambiguous facial expressions as disgust and less likely as anger.

##### Neural Correlates of Facial Emotion Processing

The processing of emotional faces is associated with a wide range of brain regions, including visual, limbic, temporoparietal, prefrontal, and subcortical areas, with some areas showing differential sensitivity to specific emotions (18). For example, the amygdala seems to be most specifically activated by fear, whereas the insula is particularly sensitive to expressions of disgust (18). A few functional magnetic resonance imaging (fMRI) studies have investigated how patients with OCD process facial emotions on a neural level, using passive or implicit viewing (38, 44) or active matching tasks (39, 52).

A study by Cannistraro et al. (38) indicates that the passive or implicit perception of faces or facial expressions in general, rather than emotional faces specifically, is associated with altered neural activity. The authors used a simple emotional faces paradigm consisting of the passive viewing of alternating blocks of fearful, happy and neutral faces. While both patients and healthy controls showed activity in left and right amygdala for fearful compared to neutral facial expressions, no between-group differences were observed for this contrast. The study did found that when contrasting all facial expressions with fixation, reactivity of the amygdala was attenuated in OCD patients compared to healthy controls.

Another study suggests altered neural processing of facial expressions of disgust in patients (44). In a backward masking paradigm that presented neutral, disgusted, and fearful facial expressions just above conscious awareness level, patients with OCD displayed increased activity in the left ventrolateral prefrontal cortex (an area involved in response inhibition and response modulation) and reduced activity in the thalamus (involved in memory, attention, and information processing) for disgusted compared to neutral expressions. Importantly, they found this effect to be driven by those patients scoring high on washing symptoms, suggesting this activity may be particularly characteristic for those who suffer from compulsions that relate to contamination concerns.

Two other studies focused on tasks that require more explicit attention to presented emotions as they involve active matching of emotional faces. Cardoner et al. (39) used a task involving the matching of a happy or fearful target face to two out of three possible emotional probe faces (happy, fearful, and angry). Results showed that matching emotional faces versus matching shapes resulted in increased activation in a distributed network of brain regions known to be involved in face processing, including the amygdala, fusiform gyrus, thalamus, and dorsolateral prefrontal cortex in OCD patients compared to controls. Patients also demonstrated significantly increased connectivity between these face-processing regions and greater activation of the right dorsolateral prefrontal cortex and the left anterior insula region for fearful compared to happy faces. In addition, the task-related activation and functional connectivity was found to be associated with symptom severity as measured by the Y-BOCS. Using a similar task, Via et al. (52) showed that matching fearful faces, compared to matching shapes, resulted in increased activation of the amygdala region in patients, as well as other regions that did not survive whole-brain level correction such as the right anterior insula cortex, premotor cortex, right orbitofrontal cortex, and right middle temporal gyrus. Amygdala activation for this contrast also significantly correlated with the severity of aggression/checking and sexual/religious dimensions. These studies suggest that when explicit emotional recognition is required, patients show increased neural reactivity in various brain regions involved in face and emotion processing, most consistently the amygdala, during the processing of fearful expressions, compared to controls.

#### Affective Prosody

Though many researchers have investigated the recognition of emotions from facial expressions, to our knowledge, only a single study has focused on the ability to identify emotions based on vocal information, i.e., prosodic intonation, in OCD (36). In this study, participants were presented with audio-recorded sentences expressing one of five basic emotions (happy, sad, surprise, fear, and anger) and were asked to identify the corresponding emotion. Results showed no significant group differences between patients and controls. The compulsion subscale of the Y-BOCS did show a significantly negative correlation with general affective prosody recognition and with the recognition of sadness specifically. These effects did however not survive Bonferroni correction. Therefore, this study indicates no deficits in the ability of individuals with OCD to recognize these five basic emotions. Yet, the sixth basic emotion of disgust, which seems especially relevant to the symptomatology of OCD, was not investigated here.

### Nonaffective Social Cues

Only few studies have investigated how individuals with OCD perceive or process nonaffective social cues, i.e., the processing of nonemotional information by others. These studies provide some initial evidence that individuals with OCD have more difficulty in perceiving social cues such as biological motion and body poses. A study focusing on the perception of biological motion, which refers to the ability to identify the movements of animate beings, showed that, compared to controls, patients were less accurate in perceiving biological motion within noise dots, and less able to discriminate between biological and nonbiological or scrambled motion (54). Their ability to perceive nonbiological motion however, was comparable to controls. A subsequent fMRI study found that during the observation of biological versus scrambled motion, patients showed aberrant activation in several brain regions, including increased activation in the right superior and middle temporal gyrus, the left inferior temporal, and fusiform gyrus, and reduced activation in the right postcentral gyrus compared to healthy controls (53). These regions have been implicated in the integration of form and motion, object and face recognition, and the visual imagery of objects (63), and the authors suggested that increased activity in these regions may reflect the exertion of additional effort or the recruitment of additional strategies in patients, whereas healthy controls have a more automatic, reflexive perception of motion. A later study investigating body and face perception, reported that patients with OCD were significantly less accurate in discriminating static pairs of bodily postures implying actions, whereas their ability to discriminate faces and chairs was unimpaired (55).

### Section Summary and Discussion: Social Cue Perception

To summarize, there is support for altered processing of both affective and nonaffective social cues in OCD, from both behavioral and neuroimaging studies. Multiple behavioral studies show specific facial emotion recognition deficits (39, 40, 49, 50), mainly with regard to expressions of disgust (40, 49, 50). Additionally, outcomes from a meta-analysis by Daros et al. (41)—including ten patient studies—also point to the presence of emotion recognition deficits in OCD, specifically for negative emotions such as disgust and, to a lesser extent, anger. Such a specific deficit in the recognition of facial expressions of disgust might represent an important marker of OCD and seems in line with studies highlighting the relevance of disgust in the symptomology of OCD, due to the role of this expression in the appraisal of potential contamination [see, e.g., (56)]. Yet, studies investigating the possible role of symptom subtype indicate no clear relation between specific symptom subtypes and facial emotion recognition deficits (40, 46, 49). It seems possible that disgust is involved in the symptomatology of OCD patients in a more general sense, as the emotion does not only convey possible contamination but also for example the violations of moral rules and interpersonal norms, to which individuals with OCD are thought to be particularly sensitive (56). Bhikram and colleagues suggest that patients with OCD learn to associate a broader range of stimuli and facial expressions with disgust due to an increased propensity to perceive them as disgusting, which might in turn decrease their ability to realistically identify stimuli expressing disgust. This is in line with the finding by Jhung et al. (42) that patients with OCD displayed a bias toward perceiving ambiguous faces as expressing disgust rather than anger. It should be noted however, that sample sizes in the studies investigating the role of subtypes were very small (N between 3 and 15), which hinders the ability to detect reliable effects.

Despite evidence for a disgust recognition deficit on a meta-analytic level, a great number of individual studies did not observe any deficits in facial emotion recognition [e.g., (36, 37, 42, 43, 46, 47)], which may suggest that deficits are associated with specific subgroups of patients or task characteristics. Although some studies show a positive relation between symptom severity and disgust recognition impairment (40, 45, 48), many studies did not and the meta-analysis by Daros et al. (41) was not able to detect such a relation based on the studies included in their review. Some studies additionally show that disgust recognition impairments are present in some but not all patients (40, 48). Interestingly, recognition of facial expressions of disgust also seem to be enhanced or restored by cognitive behavioral therapy and SSRI treatment (45, 49), suggesting that treatment status may play a role. Clearly, more research into possible moderating variables is required.

Besides initial evidence for a bias toward perceiving ambiguous faces as expressing disgust (42), individuals with OCD may be characterized by a bias to perceive facial expressions as more negatively valenced than they actually are (35). Such a bias is often also present in depression [see (64) for a review], and future studies are therefore needed to investigate to what extent the presence of depressive symptoms may account for this. Interestingly, biases toward threat-related stimuli have not been reported so far in OCD, which is remarkable given that this is commonly reported in anxiety disorders (57).

Neuroimaging studies demonstrate altered activation in various brain areas during the processing of facial emotions in OCD patients (38, 39, 44, 52), even in the absence of behavioral differences in facial emotion recognition. This seems to suggest that patients with OCD process emotional information differently, perhaps because they recruit compensatory mechanisms. Interestingly, reduced or similar amygdala activation was found in patients compared to healthy controls during the passive viewing or indirect perception of facial expressions in general (38, 44) while enhanced activation of this area was observed in tasks that required active recognition of emotional expressions (39, 52). The amygdala is involved in many different processes, and responds to a variety of emotional stimuli, but has been most consistently implicated in mediating fear and anxiety reactions, and heightened amygdala responses have often been observed in disorders of anxiety (65). Increased amygdala reactivity during situations in which OCD patients have to pay active attention to facial emotions and label or match them, and during the perception of fear specifically, therefore seems consistent with a heightened emotion or threat responsiveness, yet the finding of reduced activity during passive or indirect viewing of facial emotions deserves further exploration. In addition, patients showed altered neural activity in several other regions, such as the ACC, insula and ventro- and dorsolateral prefrontal cortex. These regions have also been implicated in neurobiological and neurocognitive accounts of the disorder [e.g., (16, 56, 66)] and increased activity in these regions may for example represent altered affective responsiveness and increased emotion regulation attempts during emotion processing (67). Moreover, altered activity in the thalamus was observed during the processing of facial emotions, an area which is thought to represent a key node in the disturbed fronto-striatal feedback loops thought to be involved in the pathogenesis of the disorder (16). Additionally, there are some indications that the specific neural alterations seem to depend on obsessive-compulsive subtype (44, 52), which highlights the importance of further elucidating the role of symptom subtypes.

The single study investigating the processing of nonfacial affective cues in OCD (36) showed no significant differences in the recognition of affective prosody between patients and healthy controls, although more severe compulsions did appear to be associated with decreased performance on the affective prosody task. Clearly, more research is needed to further explore possible deficits in the recognition of emotions from other cues than facial expressions in OCD, such as vocal, auditory or bodily cues.

There is also a scarcity of studies in the domain of nonaffective social cue perception. The few studies that do exist indicate that OCD patient seem to have difficulties identifying biological motion and body poses but not faces implying action (54, 55). Jung et al. (53) additionally showed that the perception of biological motion was associated with altered activity in several brain regions associated with the representation of visual information. These results suggest that it is possible that OCD patients already experience impairments at very basic, visual levels of social cognition.

## Mentalizing/ToM

The terms mentalizing and ToM are often used interchangeable and refer to the ability to infer the mental states of others (68). ToM is often divided in the ability to infer the feelings and emotions of others (affective ToM) and the ability to infer other people's intentions and beliefs [cognitive ToM; (69)]. ToM has been found to involve many brain regions, most consistently the temporoparietal junction extending to the superior temporal sulcus, and the medial prefrontal cortex (dorsomedial- and ventromedial prefrontal cortex), but also regions thought to be engaged in a more task-specific manner such as the precuneus, anterior temporal lobes, inferior frontal gyrus including the orbitofrontal cortex, amygdala, insula, and ACC (29, 70). Research generally distinguishes first-order (e.g., what is that person thinking)? and more complex second-order (e.g., what is he/she thinking that another person is thinking)? levels of ToM (71). A more recent division additionally separates social-cognitive and social-perceptual components (72, 73). Social-cognitive ToM involves inferring mental states of others based on their behavior, and reflects “reasoning” processes. Social-perceptual ToM, on the other hand, refers to the ability to infer other's mental states based on perceptual features. The current section will focus on studies investigating ToM abilities in OCD patients (*Mentalizing/ToM in OC*) and on the role of symptom severity and level of insight into one's own mental illness (*The Role of Symptom Severity and Level of Insight in ToM*). No studies investigating the neural correlates of mentalizing/ToM in OCD were identified. **Table 2** contains an overview of the studies discussed in this section.

**Table 2:** Overview of studies investigating theory of mind in obsessive-compulsive disorder.

Author	Method	Participants	Comorbid diagnosis?	Concurrent medication/therapy?	Task	ToM domain	Diagnosis or symptom assessment	Main results
Bozikas et al. (36)	Case-control Outpatients	OCD = 25 [32.7 ± 9.0, 10M:15F]; HC = 25 [33.4 ± 7.3, 14M:11F]	Depression (n=4), PD (n=2).	ATD without (n=11) and with (n=5) atypical antipsychotics, antipsychotics only (n=1). All patients were receiving CBT.	Fantie's Affective Cartoon Test	Social-perceptual, affective	DSM-IV, MINI/Y-BOCS	No significant differences between OCD patients and HC.
Buhlmann et al. (74)	Case-control	OCD = 35 [34.0 ± 9.1, 18M:17F]; HC = 35 [32.7 ± 11.0, 14M:21F]	MDD (n=7), panic disorder (n=2), specific phobia (n=2), AA (n=1), CTD (n=1), dysthymia (n=1), hypochondriasis (n=1).	Not reported.	Movie for the Assessment of Social Cognition	Multimodal assessment	SCID	No significant differences between OCD patients and HC.
İnanç and Altıntaş (75)	Patients only (in- and outpatients) Correlational study	OCD = 71 (subgroups: treatment resistant = 30 [32.8 ± 9.0, 8M: 22F], treatment responders = 41 [32.4 ± 9.8, 12M:29F])	Exclusion criteria included several psychiatric conditions including active schizophrenia or psychosis, acute suicidality, and substance abuse.	Not reported.	RMET	Social-perceptual, affective	SCID/Y-BOCS	Significant negative correlation between the RMET and the level of insight (p < 0.01), and between the RMET and symptom severity (p < 0.01). RMET scores were also significantly lower in the treatment-resistant group (p=0.001).
Liu et al. (76)	Case-control Outpatients	OCD = 40 [24.6 ± 4.1, 18M:22F]; HC = 38 [23.3 ± 2.7, 16M:22F]	Comorbid psychiatric disorder was considered an exclusion criterion.	Not reported.	Yoni task	First-order, second-order, cognitive + affective	SCID/Y-BOCS	OCD patients scored significantly lower than HC in second-order, affective mental state attributions (p=0.002), even after neurocognitive functioning was taken into account (p=0.023).
Mavrogiorgou et al. (47)	Case-control Outpatients	OCD = 20 [38.1 ± 10.6, 12M:8F]; HC = 20 [38.2 ± 13.0, 12M:8F]	Comorbid MDD or ADs were not considered exclusion criteria.	SSRIs (n=18), St. John's wort (n=1), SSRIs plus antipsychotic drugs (n=13). CBT was not consider an exclusion criteria.	Hinting Task (double-bluff, persuasion, mistakes, and white lies stories), faux pas test, proverb test	First-order, second-order, social-cognitive	ICD-10 and DSM-IV criteria/Y-BOCS	No significant difference between OCD patients and HC with regard to ToM tasks. However, patients with OCD performed marginally worse on the proverb task (p=0.053).
Misir et al. (73)	Case-control Outpatients	OCD = 34 [32,4 ± 10.0, 13M:21F]; HC = 30 [34,4 ± 9,7, 17M:13F].	Comorbidities not reported, but many psychiatric conditions served as exclusion criteria.	SSRI's (n=29). Therapy not reported.	DEToMI (includes first- and second-order false belief tasks, irony, metaphor and faux pas recognition tasks), RMET.	First- and second-order, social-cognitive + social-perceptual, affective	SCID/Y-BOCS	Patients' DEToMI (p=0.002) and RMET total scores (p=0.005) were significantly lower than HC. When controlled for neurocognitive functioning, between-group difference for RMET was no longer significant (p=0.087). There also was a moderate negative correlation between symptom severity and DEToMI total score (r= −0.376; p=0.026).
Pertusa et al. (77)	Case-control	OCD (n=31), AD (n=19), and HC (n=55).	GAD (n=8), PD +/- agoraphobia (n=5), SP (n=5), MDD (n=2), ED (n=2), dysthymia (n=1).	Not reported.	RMET	Social-perceptual, affective	SCID/DY-BOCS	No significant differences between OCD patients and HC.
Pino et al. (78)	Case-control	OCD = 24 [39.1 ± 12.9, 12M:11F]; HC = 23 [38.7 ± 11.9,13M:11F].	Comorbidities: axis I disorders were considered as exclusion criteria.	Not reported.	RMET	Social-perceptual, affective	SCID/Y-BOCS	No significant differences between OCD patients and HC.
Sayin et al. (79)	Case-control Outpatients	OCD = 30 [34.3 ± 11.5, 10M:20F]; HC = 30 [33.0 ± 10.6, 10M:20F].	Not reported.	ATD only (n = 18), ATD + antipsychotics, (n = 6), ATD, antipsychotics + benzodiazepines (n=6). Therapy not reported.	First- and second-order false belief tasks, hinting task, double-bluff story from “Strange Stories” set.	First- and second-order, social-cognitive	SCID/Y-BOCS	Patients scored significantly worse on the double-bluff task compared to HC (p < 0.01). Performance on double-bluff task was positively correlated with visual reproduction immediate recall (r=0.411, p <0.05) and visual reproduction-delayed recall (r=0.478, p < 0.05), while the hinting task was positively correlated with verbal memory (r=0.481, p < 0.05).
Tulacı et al. (80)	Case-control	OCD = 80 (subgroups: PI [n=24, 31.2 ± 11.3, 9M:15F], GI [n=56, 28.8 ± 9.0, 19M: 37F]); HC = 80 (no demographics provided).	Presence of comorbidities (PI: n=13, GI: n=33).	Single ATD (PI: n=8, GI: n=39), > 1 ATD (PI: n=1, GI: n=3), 1 ATD and 1 antipsychotic (PI: n=12, GI: n=6), > 1 ATD and antipsychotic (PI: n=2, GI: n=0). Therapy not reported.	First-order and second-order false-belief tasks, hinting test, Faux Pas test, double-bluff story from “Strange Stories” set, RMET.	First- and second-order, social-cognitive + social-perceptual, affective	SCID/Y-BOCS	Scores were significantly lower in patients than HC for all ToM tasks (p < 0.05). Scores were also significantly lower in the PI compared to GI group (p < 0.05). No significant differences between good insight group and HC for first- and second-order false-belief or RMET scores (p > 0.05). When comparing GI patients with HC, only faux pas, and double-bluff test scores were significantly lower in patients (p < 0.05).

ToM, Theory of mind; OCD, obsessive-compulsive disorder; HC, healthy controls; RMET, Reading the Mind in the Eyes Task; PD, panic disorder; ATD, antidepressant; K-SADS, Kiddie-Schedule for Affective Disorders and Schizophrenia; DSM, Diagnostic and Statistical Manual of Mental Disorders; MINI, Mini International Neuropsychiatric Interview; MDD, major depression disorder; AA, alcohol abuse; CTD, chronic tic disorder; SCID, Structured Clinical Interview for DSM Axis I Disorders; OCI-R, obsessive-compulsive inventory revised; SSRIs, selective serotonin reuptake inhibitors; Ads, anxiety disorders; CBT, cognitive-behavioral therapy; ICD, International Statistical Classification of Diseases; DEToMI, Dokuz Eylül Theory of Mind Index; HD, hoarding disorder; GAD, generalized anxiety disorder; SP, social phobia; ED, eating disorder; PI, poor insight; GI, good insight.

### Mentalizing/ToM in OCD

The Reading the Mind in the Eyes Task (RMET) represents a measure of affective, social-perceptual ToM, whereby individuals are required to infer emotional and mental states of others based on only the eye region of the face (81). Two studies in patients report lower RMET scores (73, 80), although after controlling for general neurocognitive functioning, between-group differences in the study by Misir and colleagues (73) were not significant anymore. Yet, two other studies report scores similar in patients and controls (77, 78).

Other studies focused on more social-cognitive aspects of ToM in OCD. Sayın, Oral, Utku, Baysak, and Candansayar (79) used a number of different tasks. An adapted version of the cartoon picture story based on Brüne (82) was used to assess first- and second-order false beliefs. A story of the so-called hinting task (83, 84) was used to assess the ability to infer real intentions behind indirect statements. To assess more advanced, “third-order” ToM (e.g., he knows they think he will lie), the double-bluff story from the set of “Strange Stories” was used (85), which asks participants to identify why a character of the story said something that was not meant literally. Although patients performed worse on all ToM tasks, the difference with controls was significant only for the double-bluff task, which they found to be associated with reduced memory capacity: performance on this task was positively correlated with both immediate and delayed recall on a visual reproduction task. Tulacı et al. (80) employed the same tasks along with a faux pas test (86) and demonstrated significant group differences, with patients performing worse on all tasks. Misir et al. (73) also showed significant social-cognitive ToM deficits in patients compared to controls in all measures of a test battery called the Dokuz Eylül ToM Index (DEToMI), which remained significant after controlling for general neurocognitive functioning. The DEToMI consists of a series of verbal or visual tasks assessing social-cognitive aspects of ToM and includes first- and second-order false belief tasks, as well as irony, metaphor, and faux pas recognition tasks (73). In contrast, Mavrogiorgou et al. (47) found no significant impairments compared to controls on the hinting task, multiple sets from “Strange Stories” nor on the faux pas test. The authors did find a marginally significant deficit on a proverb test (87), which assesses the ability to recognize the hidden meaning behind indirect speech and which has been found to be strongly related to ToM (88). Thus, most but not all studies show deficient social-cognitive ToM in OCD patients.

Liu et al. (76) specifically compared affective and cognitive components of ToM using the so-called Yoni task (89). In this task, a cartoon face was presented in the middle of the screen with four colored pictures in each corner of the screen. Participants had to identify the picture that the cartoon was referring to based on an incomplete sentence at the top of the screen and cues such as the eye gaze and expression of the cartoon face and the facial expressions of the corner images. The study demonstrated impairments in OCD patients specifically on second-order, cognitive levels of ToM, which remained significant after controlling for general neurocognitive abilities, while first-order and affective levels of ToM were not significantly different from controls. A single study by Buhlmann, Wacker, and Dziobek (74) employed a multimodal task called the Movie for the Assessment of Social Cognition (90) to assess general ToM skills in OCD patients. In this task, participants watched a short movie and were instructed to answer questions about the characters' thoughts, intentions and emotions at set time points during the movie. No differences between OCD patients and controls were found, suggesting that patients with OCD do not show impairments during more integrated assessments of ToM.

### The Role of Symptom Severity and Level of Insight in ToM

İnanç and Altıntaş (75) observed a negative relation between symptom severity and RMET performance in patients, while Misir and colleagues (73) observed a moderate negative correlation between symptom severity and DEToMI total score. Yet, other patient studies did not demonstrate significant relations between symptom severity and ToM (47, 76, 78, 79). There is however evidence to suggest that the extent to which patients are aware of the irrationality of their obsessions and/or compulsions, i.e., their level of insight, is related to ToM abilities (73, 75, 80). Tulacı et al. (80) found significant negative correlations between insight level and all ToM tasks, with ToM performance significantly lower in patients with poor compared to good insight. Interestingly, patients with good insight did not differ from healthy controls on the RMET and first- and second-order false belief task, but did score significantly lower on the double bluff, faux pass and hinting task. Misir et al. (73) also reported a negative correlation between the level of insight and the DEToMI total score. İnanç and Altıntaş (75) specifically investigated the role of insight within a sample of treatment-resistant and treatment-responding patients. They found a significant negative correlation between RMET performance and level of insight. RMET scores were also significantly lower in the treatment-resistant group. Thus, these studies suggest that ToM may be especially impaired in those OCD patients with poor illness insight, and to a lesser extent in patients with good insight.

### Section Summary and Discussion: Mentalizing/ToM

In summary, there is some evidence for deficient mentalizing or ToM in OCD. Some of these studies find deficits in both affective and cognitive ToM (73, 80) whereas in other studies deficits are limited to (social-)cognitive and higher-order domains (76, 79). Yet other studies, however, show no clear deficits (36, 47, 74, 77, 78). The observed ToM deficits seem to depend in part on more general cognitive abilities (73, 79), which is unsurprising as ToM tasks draw upon general cognitive and verbal abilities to a much greater extent than lower-level processes such as emotion recognition [see, e.g., (91)]. These studies thus indicate that the cognitive deficits that patients with OCD experience may also impact on social cognitive abilities such as ToM. However, ToM deficits in OCD do not seem to be explained by more general cognitive deficits alone (73, 76), highlighting the importance of investigating social cognition in the disorder as a separate construct.

While most studies do not indicate a significant relation between ToM and symptom severity (47, 76, 78, 79), level of illness insight of patients does appear to be an important moderator of ToM deficits (73, 75, 80). Poor insight in OCD is associated with several clinical characteristics, such as higher comorbidity rates, specifically depression and schizophrenia spectrum disorders, poorer treatment response, more severe symptoms, and longer illness duration (92, 93). Notably, obsessive-compulsive symptoms are highly prevalent in schizophrenia and patients with first-episode psychosis with prevalence rates up to 64% (94), and the presence of these symptoms have been associated with poorer social cognitive abilities in patients with schizophrenia, specifically for higher-order ToM (95). Approximately 22%–25% of patients are characterized by poor insight (92, 93). As such, it seems possible that these patients represent a subgroup of OCD with greater ToM disturbances. However, more general factors related to poor insight such as poorer global, cognitive, and intellectual functioning may also play a role (94).

To our knowledge, no studies have investigated the neural correlates of ToM in relation to OCD. Given the observed deficits in ToM inferences, regions involved in ToM such as the temporoparietal junction and the medial prefrontal cortex may be affected. Furthermore, several brain regions implicated in the psychopathology of OCD [see, e.g., (16)] have been linked to ToM as well. For example, it has been suggested that more affective or implicit ToM assessments involve regions such as the orbitofrontal cortex, (dorsal) ACC, and insula, whereas cognitive and explicit assessments depend on brain areas related to more general cognitive resources such as the rostral ACC and medial and lateral PFC (29). Future studies may provide important insights into the underlying neural mechanisms of disturbed ToM inferences.

## Experience Sharing and Empathy

Experience sharing refers to the vicarious experience and brain activity that is triggered by observing behavior of others. Green et al. (4) divide this concept in “motor resonance” and “affect sharing.” Motor resonance is defined as the functional correspondence between the motor state in others and the self and is believed to represents a bottom-up process involving the so-called mirror neuron system [MNS; (4)]. This system consists of a group of neurons that are thought to be involved in the recognition and understanding of others actions by imitating or “mirroring” the actions or behaviors performed by others as they are activated by both the execution and observation of actions (95). It involves a network of brain regions including the inferior frontal gyrus, dorsal, and ventral premotor cortex, and the inferior and superior parietal lobule as well as other regions depending on sensory modality (96). For example, the execution and observation of emotional expressions demonstrates vicarious activity in regions such as the insula, amygdala, and cingulate gyrus (96).

The second aspect of experience sharing is “affect sharing,” which refers to the observation of emotional expressions in others and the corresponding experience of these emotions as well as the activation of emotion-related brain areas in the self (4). Affect sharing is thought to represent a bottom-up process depending on the coupling of perception and action which possibly involves the MNS, and is considered a crucial subcomponent of empathy (97, 98). Empathy is considered a multifaceted construct including both bottom-up affect sharing processes as well as more top-down executive processes such as perspective taking skills and emotion regulation, which are mostly thought to involve prefrontal brain regions (99, 100). Many researchers also distinguish between affective empathy (the ability to share others' emotional states) and cognitive empathy [the ability to understand others' emotions; see, e.g., (69)]. By this definition, cognitive empathy is equated with affective ToM. Yet other researchers narrow down the concept of empathy to the isomorphic state (knowingly) elicited by the affective state of others [e.g., (101)]. The following section will focus on motor resonance (*Motor Resonance*) and affect sharing and empathy (*Affect Sharing and Empathy*). Research on emotion regulation, which constitutes a critical subcomponent of empathy, will be discussed below in the section *Emotion Experience and Regulation*. **Table 3** contains an overview of the studies discussed in this section.

**Table 3:** Overview of studies investigating experience sharing and empathy in obsessive-compulsive disorder.

Domain	Author	Method	Participants	Comorbid diagnosis?	Concurrent medication/therapy?	Task/questionnaire	Subdomain	Diagnosis/symptom assessment	Main results
*Empathy*									
	Fontenelle et al. (102)	Case-control	OCD = 53 [39.3 ± 13.8, 29M:36F]; HC = 53 [35.5 ± 13.0, 24M:46F]	MDD (n=19), SP (n=3), DD (n=3).	SSRIs (n=42), benzodiazepine (n=21), antipsychotic (n=17). Therapy: CBT (n=17).	IRI	Cognitive empathy (PT and FT) + affective empathy (EC and PD)	SCID/OCI-R	Compared to HC, patients showed higher levels of EC (p=0.006) and PD (p < 0.001). Within patients, hoarding symptoms correlated with EC (r=0.39; p < 0.001), FT (r=0.36; p < 0.01), and PD (r=0.39; p < 0.001). After adjusting for covariates, only the association between hoarding and FT remained (r=0.41; p < 0.001).
	Kang et al. (103)	Case-control	OCD = 107 [27.5 ± 9.22, 72M:35F]; HC = 130 [26.0 ± 4.8, 82M/48F]	MDD (n=20), SP (n=5), BDD (n=20, panic disorder (n=1).	All patients were taking medications. Therapy not reported.	IRI	Cognitive empathy (PT and FT) + affective empathy (EC and PD)	SCID/Y-BOCS	Patients with OCD showed significantly lower PT (p=0.003) and higher PD (p=0.001) compared to HC. PD correlated significantly with forbidden thoughts symptoms (r=0.254, p=0.017) after correcting for gender, anxiety and depression levels.
	Pino et al. (78)	Case-control	OCD = 24 [39.1 ± 12.9, 12M:11F]; HC = 23 [38.7 ± 11.9,13M:11F]	Comorbid disorders were considered as exclusion criteria.	Not reported.	BES, EQ, EAT	Cognitive (BES cognitive, EQ, EAT) and affective empathy (BES affective)	SCID/Y-BOCS	OCD patients scored lower than controls on the EQ (p < 0.001), cognitive subscale of the BES (p=0.020) and attribution of negative emotions except disgust in the EAT (ps <0.005). There also was a positive relation between the cognitive BES subscale and Y-BOCS obsessions (r=−0.423, p=0.002) and compulsions (r=−0.420, p=0.003).subscales. No differences were found between patients and HC on the affective empathy subscale of the BES.
*Motor resonance*									
	Kim et al. (54)	Case-control Outpatients	OCD = 20 [24.3 ± 6.2, 12M:8F]; HC = 16 [23.2 ± 5.8, 11M:5F]	Not reported.	Sertraline (n=4), citalopram (n=6), fluoxetine (n=5), fluvoxamine (n=2), risperidone (n=5), olanzapine (n=1), clonazepam (n=14), valproic acid (n=1), and lamotrigine (n=1). Therapy not reported.	Biological motion detection and discrimination tasks	Biological motion perception	DSM-IV criteria/Y-BOCS	Patients found it more difficult to detect biological motion within noise dots (p=0.003) and to discriminate biological motion from scrambled motion (p=0.034), whereas their ability to perceive nonbiological global motion and static global form was comparable to HC.
	Jung et al. (53)	Case-control fMRI	OCD = 15 [23.4 ± 4.7, 12M:3F]; HC = 15 [25.67 ± 3.46, 9M:6F]	Comorbid axis I diagnoses were considered exclusion criteria.	Monoamine oxidase inhibitors (n=2), SSRI + antianxiety (n=3), SSRI + antianxiety + anti-psychotics (n=3). Therapy not reported.	One-back task with biological and scrambled motion	Biological motion perception	SCID for DSM-II/Y-BOCS	Compared to HC, patients exhibited increased activation in the right superior and middle temporal gyrus, the left inferior temporal and fusiform gyrus and reduced activation in the right postcentral gyrus (p < 0.001, uncorrected).
	Rounis et al. (104)	Case-control Outpatients	OCD = 24 [37.9 ± 14.7; 14M:10F] HC = 22 [37.4 ± 13.5; 12M:10F]	Comorbid psychiatric disorders were considered exclusion criteria.	SSRI (n=15) and SSRI + antipsychotic (n=4).	Meaningless gesture imitation task, extracted from the Birmingham Cognitive Screen	Action imitation	MINI/Y-BOCS	Scores on hand and finger imitation gestures were significantly lower for patients compared to HC (p=0.001). There were no significant correlations of imitation scores with the Y-BOCS.
	Shin et al. (55)	Case-control Outpatients	OCD = 54 [25.0 ± 6.5, 32M:22F]; HC = 42 [23.4 ± 4.6, 32M:10F]	Comorbid axis I diagnoses were considered exclusion criteria.	Medication-naïve (n=24), medication-free for 4 weeks (n=30). Therapy not reported.	Body and face discrimination task	Recognition of faces and bodies	SCID	Compared to HC, patients were less accurate in discriminating human bodily postures (p < 0.001), but not in discriminating faces or chairs.

OCD, obsessive-compulsive disorder; HC, healthy controls; MDD, major depressive disorder; SP, social phobia; DD, dysthymic disorder; SSRIs, selective serotonin reuptake inhibitors; CBT, cognitive-behavioral therapy; IRI, Interpersonal reactivity index; PT, Perspective Taking; FT, Fantasy; EC, Empathic Concern; PD, Personal Distress; SCID, Structured Clinical Interview for DSM Axis I Disorders; OCI-R, Obsessive-compulsive Inventory Revised; BDD, Body dysmorphic disorder; BES, Basic Empathy Scale; EQ, Empathy Quotient; EAT, Emotion Attribution Task; Y-BOCS, Yale–Brown Obsessive-Compulsive Scale; Diagnostic and Statistical Manual of Mental Disorders; MINI, Mini International Neuropsychiatric Interview.

### Motor Resonance

Although no studies have directly investigated how the actions of others are represented in the brain of patients with OCD, there is some indirect evidence to suggest that patients with OCD may show deficient motor resonance. A study by Rounis, Banca, and Voon (104) for example showed that patients with OCD scored significantly lower than healthy controls on a task that required them to imitate meaningless hand and finger gestures performed by an experimenter. In addition, previously discussed studies (*Nonaffective Social Cues*) on the recognition of biological motion (53, 54) and body poses implying action (55) may likewise indicate a deficiency in representing the actions of others in the brain. Besides behavioral reports of impairments in motion or action recognition (54, 55), the study by Jung et al. (53) showed that patients demonstrated increased activity in several brain regions that are thought to be part of the MNS during the perception of biological motion, and have proposed that this activation may reflect increased effort or neural inefficiency of this system. However, since their study concerned moving black dots rather than real human beings performing actions, direct evidence for altered motor resonance and MNS functioning in OCD is still missing.

### Affect Sharing and Empathy

Current measures of affect sharing and empathy in OCD are limited to self-report questionnaires such as the Interpersonal reactivity index [IRI; (105)]. The IRI represents a widely used measure of empathy containing four subscales, of which two scales measure affective components of empathy (empathic concern and personal distress) and two scales measure cognitive components (perspective taking and fantasy). Empathic concern refers to feelings of concern and sympathy for others, whereas the personal distress scale focuses on self-oriented feelings of anxiety and distress intense interpersonal situations. Empathic concern is thought to promote prosocial behavior toward others (105), whereas the experience of interpersonal distress is often considered maladaptive, and has been found to be elevated in mood and anxiety disorders (106). The perspective taking subscale refers to one's more cognitive tendency or ability to spontaneously adapt the viewpoint of others, whereas the fantasy scale measures the tendency to identify oneself with fictitious characters in books, movies, or plays.

Using the IRI, Fontenelle et al. (102) demonstrated that patients with OCD displayed greater self-reported levels of empathic concern and personal distress compared to healthy controls. Within patients, higher neutralizing and hoarding symptoms as measured by the obsessive-compulsive inventory–revised (OCI-R) were associated with high scores on the fantasy dimension. Patients with higher symptoms of checking, ordering, washing, and hoarding also showed more empathic concern, whereas all symptom dimensions were related to higher personal distress. However, after correcting for comorbid depression and anxiety, only the relation between hoarding symptoms and fantasy remained. In another sample of OCD patients, Kang, Namkoong, Yoo, Jhung, and Kim (103) showed increased personal distress and decreased perspective taking compared to healthy controls, with no differences for empathic concern or fantasy. When taking symptoms of depression and anxiety into account, the personal distress scale was also positively related to the forbidden thoughts dimension of the Y-BOCS measure of OCD symptoms, which refers to the presence of obsessions related to aggression, sex, and religion. These studies suggest that patients may be characterized by increased affective levels of empathy, especially with regard to empathic distress, and possibly decreased cognitive empathic abilities, as indicated by poorer perspective taking skills. However, these differences may be in part explained by comorbid levels of anxiety and depression, rather than specific symptom dimensions of OCD, as correlations with specific symptom dimensions often disappeared after including depression and anxiety levels as covariate.

In a study using different empathy measures (78), patients with OCD had lower scores than controls on the cognitive empathy subscale of the Basic Empathy Scale [BES; (107)] and on the Empathy Quotient (108), a questionnaire focusing mostly on cognitive empathy. Pino et al. (78) also showed a negative relation between scores on the cognitive BES subscale and the presence of obsessions and compulsions (as assessed by the Y-BOCS). Participants in this study also performed an emotion attribution task, in which the ability to identify the emotions of other's based on short stories was assessed (109). Here, patients scored lower than controls on the attribution of all negative emotions except disgust. However, Pino et al. (78) found no differences were compared to controls on the affective empathy subscale of the BES. Thus, this study indicates that OCD patients are characterized by specific deficits in cognitive, but not affective components of empathy.

### Section Summary and Discussion: Experience Sharing and Empathy

Few studies have been conducted on experience sharing and empathy in patients with OCD. There are some indirect indications that patients with OCD may show deficient motor resonance or impaired MNS functioning as they have been shown to display poorer imitation of other's actions (104), impaired recognition and neural processing of biological motion (53, 54) and deficient perception of body poses implying actions (55), yet direct evidence for altered motor resonance from neuroimaging studies are missing. Likewise, there are no neuroimaging or experimental studies on affect sharing in patients with OCD. Evidence from self-report questionnaires does indicate that patients experience a heightened affective responsiveness to emotions of others (102, 103) or a similar emotional congruence with others compared to controls (78). Increased affective distress may be linked to more general levels of anxiety or depression, as most correlations with specific symptom dimensions did not remain after taking this into account. With regard to more top-down, cognitive aspects of empathy, some studies indicate a decreased self-reported ability to understand the emotions of others (78, 103), with scores on the emotion attribution task providing more experimental evidence for this (78). These findings seems in line with previously discussed experimental studies on affective ToM showing a decreased ability to identify the emotions of others in patients using the RMET (73, 80), which has also been considered as an index of cognitive empathy. Importantly however, research on experience sharing and empathic functioning in OCD is still in its infancy. Future studies using experimental as well as neuroimaging methods may shed more light on the specificity and origin of empathic alterations in the disorder.

## Emotion Experience and Regulation

The term “emotion experience” refers to the emotion reactions (on either a subjective, observable, or neurophysiological level) that individuals experience in response to positive or negative stimuli (4). The ability to exert control over how and when these emotions are experienced and expressed is called emotion regulation (67). Whereas emotional reactivity is known to involve the dorsal anterior cingulate, insula, amygdala, and periaqueductal grey (PAG), explicit or conscious (top-down) regulation of emotion is associated with brain activity in the dorso- and ventro lateral prefrontal cortex, (pre)supplementary motor area and parietal cortex. Emotion regulation can however also be an automatic (bottom-up) process, and more implicit or unconscious emotion regulation has been linked to the ventral anterior cingulate and the ventromedial prefrontal cortex (67).

Given that OCD was until recently defined as an anxiety disorder, it has long been recognized that abnormal experience and regulation of emotions plays a crucial role in the symptomatology of OCD [see, e.g., (110)]. It has even been argued that the mental and behavioral compulsions that characterize OCD patients represent a maladaptive coping or emotion regulation mechanism of dealing with aversive and unwanted emotions triggered by obsessional thoughts (111). However, emotional disturbances may also importantly impact how we deal with social situations. For example, an influential framework by Decety and Meyer (100) suggests that emotion regulation is an important cognitive skill which helps control one's own arousal or distress. Individuals who become overaroused by other's distress due to problems with emotion regulation, might therefore be unable to deal with others emotions in a prosocial or adaptive fashion due to the cognitive resources that are used up too regulate their own emotions (112). Emotion regulation is thus considered a crucial subcomponent for adaptive empathic responding. Given that the way we experience and regulate our emotions is of critical importance for successful social interaction, the following section will describe existing research on the experience (*Emotion Experience*) and regulation (*Emotion Regulation*) of emotions in patients with OCD. **Table 4** contains an overview of the studies discussed in this section.

**Table 4:** Overview of studies investigating emotion experience and emotion regulation in obsessive-compulsive disorder.

Domain	Author	Methods	Participants	Comorbid diagnosis?	Concurrent medication/therapy	Task/questionnaire	Emotions assessed/stimulus-type	Diagnosis/symtom assessment	Main results
*Social emotion experience*									
	Basile et al. (113)	Case-control fMRI	OCD = 13 [37.0 ± 11.1; 10M:3F] HC = 19 [26.2 ± 2.1; 11M:8F]	Comorbid MDD and ADs not considered as exclusion criteria.	SSRI or tricycles (n=6). Therapy: CBT (n=9).	Guilt-judgement task	Deontological guilt, altruistic guilt, anger, sadness	DSM-IV-TR criteria/PI/Y-BOCS	Compared to HC, OCD patients felt significantly more guilt in both the deontological guilt (p < 0.02) and altruistic guilt condition (p < 0.009). When experiencing guilt compared to nonmoral emotions (anger and sadness), patients exhibited reduced activation in the ACC, superior and medial frontal gyri (p < 0.001).
	Bersani et al. (114)	Case-control Outpatients	OCD = 10 [40.22 ± 13.49; 5M:5F] Schizophrenia = 10 [40.88 ± 12.97; 5M:5F] HC = 10 [40.20 ± 10.49; 5M:5F]	Comorbid axis I diagnoses were considered exclusion criteria.	OCD: clomipramine (n = 7), fluvoxamine (n = 4) sertraline (n = 1), escitalopram (n = 1), citalopram (n = 1), valproic acid (n = 3), gabapentin (n = 1), alprazolam (n = 3), lorazepam (n = 1), and zolpidem (n = 1). Schizophrenia: paliperidone (n = 3), aripiprazole (n = 4), olanzapine (n = 1), quetiapine (n = 1), clozapine (n = 1), risperidone (n = 1), valproic acid (n = 4), and lithium (n = 1). Therapy not reported.	Emotion-eliciting videoclip of social scenarios while facial activity was videotaped	Amusement, fear, surprise, anger, sadness, disgust, neutral	SCID/BPRS/Y-BOCS	Compared to HC, OCD patients showed significantly less concordant responses (p=0.004), more discordant responses (p=0.003) and less facial expressions (p < 0.001). No differences were found between OCD and schizophrenia patients.
	Fontenelle et al. (115)	Case-control fMRI	OCD = 18 [34.8 ± 11.5; 11M:7F] HC = 18 [32.4 ± 9.2; 11M:7F]	Borderline and antisocial personality disorders, alcohol or substance abuse and suicidality were considered as exclusion criteria.	Almost all OCD were medicated with SSRI, with the exception of one with SNRI. Also with: antipsychotics (n=7), benzodiazepines (n=6), tricyclic antidepressant (n=1), topiramate (n=1), memantine (n=1). Therapy not reported.	Moral sentiments association task	Guilt, compassion, anger, disgust, neutral	SCID/Y-BOCS/DOCS	During guilt provocation, OCD showed higher activity in postcentral gyrus and reduced activity in angular gyrus compared to HC (p < 0.005). During compassion provocation, OCD showed higher activity in dorsal anterior cingulate compared to HC (p < 0.005). During anger provocation, OCD showed higher activity in caudate nucleus, paracingulate and precentral gyri, and reduced activity in angular gyrus compared to HC (p < 0.005). During disgust provocation, OCD showed higher activity in medial frontal/paracingulate cortex and decreased activity in left NAcc compared to HC (p < 0.005). The combined emotion analysis revealed that OCD showed higher activity in lingual gyrus and decreased activity in left NAcc and middle temporal gyri compared to HC.
	Hennig-Fast et al. (116)	Case-control fMRI	OCD = 20 [31.10 ± 8.58; 10M:10F] HC = 20 [29.70 ± 4.75; 10M:10F]	None of the participants received any additional comorbid axis I diagnoses.	Not reported.	Imaginative emotion-inducing task	Shame, guilt, neutral	DSM-IV criteria/SCID/Y-BOCS	Patients reported higher levels of shame and guilt on the questionnaires administered, but not during the experimental task, compared to HC. In the shame compared to neutral condition, OCD showed increased activation in bilateral middle temporal gyrus, left uncus, left parahippocampal gyrus and hypothalamus, and decreased activation in middle frontal gyrus and inferior parietal lobule, compared to HC (p < 0.001). In the guilt compared to neutral condition, OCD showed increased activation in left superior frontal gyrus, right precentral gyrus, bilateral cingulate gyrus, right superior temporal gyrus and right sub-gyral region, and decreased activation in left anterior cingulate, compared to HC (p < 0.001). In the OCD group, Y-BOCS scores correlated positively with activation of left middle, bilateral superior, left medial frontal gyri, bilateral parahippocampal gyrus and left posterior cingulate, and negatively with activation of precuneus during shame condition. Y-BOCS scores correlated positively with activation of left middle frontal gyrus and temporo-parietal junction during guilt condition.
	Mergl et al. (117)	Case-control Outpatients	OCD = 34 [35.8 ± 11.5; 19M:15F] HC = 34 [37.5 ± 13.1; 19M:15F]	Not reported.	Studied in unmedicated state and after a 10-week treatment with the SSRI sertraline and semi-standardized behavioral therapy.	Emotion-inducing (humorous) videoclip of Mr. Bean	Laughter	Y-BOCS/CGI	Compared to HC, patients showed slower initial velocities of involuntary facial movements in left eye (p=0.007), right eye (p=0.014), left angle of the mouth (p=0.003) and right angle of the mouth (p=0.013). Patients and HC rated the videoclips as equally humorous, however the frequency of laughing reactions was significantly lower in OCD (p < 0.001). Higher Y-BOCS scores were associated with lower laughing frequencies (p=0.011).
	Valeriani et al. (118)	Case-control Outpatients	OCD severe = 10 [40.61 ± 6.12; 5M:5F] OCD mild-moderate = 11 [37.77 ± 8.21; 5M:6F] HC = 15 [41.71 ± 12.53; 7M:8F]	Comorbid axis I diagnoses were considered exclusion criteria.	Valproate (severe=6, mild=3), SSRI (severe=9, mild=10), aripiprazole (severe=4), benzodiazepines (severe=7, mild=7), clomipramine (severe=6, mild=1).	Emotion-eliciting videoclip of social scenarios	Surprise, fear, happy, disgust, anger, sadness	SCID/Y-BOCS	HC reported more concordant responses compared to both severe (p < 0.01) and mild OCD (p=0.02). Severe OCD showed less concordant facial expressions compared to mild OCD (p=0.03) and HC (p < 0.01), and mild OCD showed less concordant facial expressions compared to HC (p < 0.01). Compared to mild, severe OCD showed significantly poorer performance in response to happiness- and disgust-eliciting videoclips.
*Nonsocial emotion experience*									
	Admon et al. (119)	Case-control Outpatients fMRI and DTI	OCD = 13 HC = 13	None of the participants met criteria for additional Axis I disorders. 3 patients met criteria for Axis II cluster A personality disorders. 3 patients had a history of MDD and 3 of social phobia.	All patients were treated with serotonergic agents (sertraline, paroxetine, escitalopram, clomipramine), 4 patients received in addition a low-dose antipsychotic agent (risperidone, haloperidol). Therapy not reported.	Interactive risky choice game	Threat, reward	DSM-IV criteria/SCID-P/Y-BOCS	Compared to HC, patients chose significantly fewer nonmatch risky choices (p=0.02). OCD showed higher activation of the amygdala in response to threat (p=0.02) and lower activation of the NAcc in response to reward compared to HC (p=0.02). Amygdala-dACC (p=0.03) and NAcc-OFC (p=0.01) were more weakly functionally connected in patients compared to HC, and stronger functional connections between these regions were related to lower severity of OCD symptoms.
	Choi et al. (120)	Case-control fMRI	PG = 15M [27.93 ± 3.59] OCD = 13M [24.92 ± 6.92] HC = 15M [26.60 ± 4.29]	OCD: tic disorder (n=1), OC personality disorder (n=1), schizotypal personality disorder (n=1).	Not reported.	Monetary incentive delay task	Reward, loss-avoidance, neutral	SCID/Y-BOCS	No statistically significant differences in BOLD response between HC and OCD during anticipation of gain. During anticipation of loss, OCD showed increased activation in the anterior insula, putamen and caudate nucleus compared to HC (p < 0.005 uncorrected).
	Figee et al. (121)	Case-control Outpatients fMRI	OCD = 18 [34 ± 8.3; 5M:13F] HC = 19 [32 ± 6.6; 6M:13F]	MDD (n=2), additional disorders on Axis I (n=4), OC personality disorder (n=2).	SSRI (n=5), tricyclic antidepressant (n=3), combined noradrenergic and serotoninergic antidepressant (n=1).	Monetary incentive delay task	Reward, no reward	MINI/Y-BOCS	Compared to HC, OCD showed reduced activation of the NAcc (bilateral) and the left insula during anticipation of monetary gain (p < 0.05 corrected). No statistically significant correlations were found between Y-BOCS scores and BOLD responses during reward anticipation.
	Hauser et al. (122)	fMRI Adults and children	OCD = 33 [23.4 ± 9.5; 21M:12F] HC = 34 [24.5 ± 11.2; 13M:21F]	MDD (n=3), panic disorder with agoraphobia (n=2), social phobia (n=4), specific phobia (n=4), GAD (n=2), body dysmorphic disorder (n=1), pain disorder (n=1), AN (n=2), ADHD (n=2), CD (n=1), other childhood emotional disorders (n=2), chronic tic disorder (n=1).	SSRI (n=13), neuroleptics (n=4), SSNRI (n=3), benzodiazepine (n=2), levothyroxin (n=2), NaSSA (n=1), anticholinergics (n=1), tricyclic antidepressant (n=1).	Probabilistic reversal learning task	Reward, punishment	SCID or K-SADS-PL/Y-BOCS or CY-BOCS/DSM-5 criteria	OCD showed increased reward prediction error-related activation in the ACC and right putamen (p < 0.05 corrected), also after controlling for age. Neither ACC nor putamen correlated with Y-BOCS total, obsessions subscale or compulsion subscale.
	Jung et al. (123)	Case-control Outpatients fMRI	OCD = 20 [25.70 ± 6.99; 13M:7F] HC = 20 [24.75 ± 3.68; 13M:7F]	Tic disorder (n=1), OC personality disorder (n=2), schizotypal personality disorder (n=1).	Medication-naïve (n=15), medication-free for 4 weeks (n=5).	Monetary incentive delay task	Gain, loss, neutral	SCID/Y-BOCS	During gain anticipation, there were no statistically significant differences between OCD and HC. During loss anticipation, OCD showed reduced activation of lateral PFC including the superior frontal cortex and postcentral cortex, and reduced activation of anterior insula, compared to HC (p < 0.001 uncorrected). In the gain outcome contrast, patients showed increased activation of putamen, precentral cortex, posterior insula, ACC and cerebellum compared to HC (p < 0.001). In the loss avoidance contrast, patients showed increased activation of ventral striatal, midbrain, superior temporal cortex and inferior parietal cortex compared to HC (p < 0.001). Ventral striatal activation in patients was significantly correlated with Y-BOCS (p=0.045).
	Jung et al. (124)	Case-control Outpatients Functional connectivity analysis	OCD = 19 [25.84 ± 7.15; 12M:7F] HC = 18 [24.83 ± 3.88; 11M:7F]	Not reported.	Medication-naïve (n=15), medication-free for 4 weeks (n=4).	Monetary incentive delay task	Gain, loss, neutral	SCID	During gain anticipation, OCD showed increased functional connectivity of the NAcc with the posterior insula and occipital regions, and reduced functional connectivity of the NAcc with the left amygdala positioned adjacent to the anterior insula, middle frontal cortex and midbrain (p < 0.01 corrected). During loss anticipation, OCD showed increased functional connectivity of the NAcc with the occipital cortex and reduced functional connectivity of the NAcc with the bilateral amygdala compared to HC (p < 0.01 corrected). OCD patients' overall symptom severity was positively correlated with functional connectivity between NAcc and medial OFC and negatively correlated with functional connectivity between NAcc and lateral OFC (p < 0.001).
	Kaufmann et al. (125)	Case-control Outpatients fMRI	OCD = 19 [34.8 ± 11.0; 8M:11F] HC = 19 [34.9 ± 11.8; 8M:11F]	Affective disorder (n=7), phobic disorders (n=3), impulse control disorder (n=1), personality disorder (n=3).	Clomipramine (n=1), venlafaxine (n=1), clomipramine + paroxetine (n=1). None of the patients took benzodiazepines within 4 weeks before the scanning session. Patients were currently under treatment (CBT).	Monetary incentive delay task	Reward, loss-avoidance, neutral	DSM-IV criteria/SCID/Y-BOCS/OCI-R	OCD showed fewer delayed responses in loss-avoidance than in reward trails, whereas the opposite was true in HC (p=0.05). No statistically significant differences were found in activation of brain regions of the reward circuitry between HC and OCD. Patients showed higher activation of superior/medial frontal and cingulate region in loss-avoidance condition compared to HC, but less activation in reward condition (p=0.018). Y-BOCS ratings did not correlate with BOLD responses.
	Koch et al. (126)	Case-control fMRI	OCD = 44 [32.7 ± 9.3; 17M:27F] HC = 37 [32.0 ± 8.0; 15M:22F]	MDD (n=16), AD (n=1), MDD + AD (n=5), personality disorder (n=2), impulse control (n=1).	SSRI (n=20), SNRI (n=4), tricyclic antidepressant (n=4), benzodiazepines (n=1), atypical antipsychotic (n=1). Therapy not reported.	Monetary reward task	Reward, punishment	DSM-IV criteria/Y-BOCS	No activation differences between HC and OCD in punishment trials. In reward trials, patients showed reduced activation in the frontal cortex bilaterally and the posterior cingulate extending into the left precuneus (p < 0.05 corrected). Patients showed a significantly increased connectivity between the left PCC/precuneus and the left vmPFC and the right PCC, compared to HC (p < 0.05 corrected). No significant correlation between Y-BOCS and connectivity patterns were found.
	Murray et al. (127)	Case-control fMRI	OCD = 18 [35.6 ± 10.1; 11M:7F] HC = 18 [32.1 ± 6.5; 15M:3F]	Comorbid axis I diagnoses were considered exclusion criteria.	Most patients were taking SSRIs.	Probabilistic learning task	Reward, punishment, neutral	DSM-IV-TR criteria/SCID	During negative prediction error processing, OCD showed higher activation of ACC compared to HC (p=0.006). During positive prediction error processing, OCD showed increased activation of NAcc compared to HC (p=0.031). There were no correlations between Y-BOCS scores and either ACC or NAcc activation.
	Remijnse et al. (128)	fMRI	OCD = 20 [34 (19-54); 5M:15F] HC = 27 [32 (22-53); 8M:19F]	PTSD (n=1), panic disorder (n=2), GAD (n=4), SAD (n=4), opioid abuse in sustained full remission (n=1), Tourette disorder (n=1).	Patients were free from psychotropic medication for at least 2 weeks, and in case of fluoxetine or antipsychotic medication for at least 1 month.	Probabilistic reversal learning task	Positive, negative, neutral feedback	SCID/Y-BOCS/PI-R	Compared to HC, OCD patients showed reduced activation of in lateral and medial orbitofrontal cortex (ps < 0.005) during reward processing.
	Riesel et al. (13)	Meta-analysis EEG	OCD = 1007 HC = 1100	Not reported.	Not reported.	Performance monitoring tasks	Not applicable.	Y-BOCS	Compared to HC, patients showed a robust increase of ERN in conflict-response tasks (p < 0.001), that was not modulated by symptom severity, depressive symptoms, medication and age.
	Thorsen et al. (129)	Meta-analysis Case-control fMRI, PET or SPECT	OCD = 571 [33.44 ± 5.91] HC = 564 54.35% of subjects were males	Comorbidities: anxiety and mood disorders in some studies.	68% of studies included medicated patients. Studies with treated patients were excluded.	Emotional processing tasks	E.g., fear, disgust, neutral, distress, urges to ritualize	Y-BOCS	Compared to HC, patients showed significantly increased activation in the right OFC extending into the sgACC and vmPFC, right putamen, bilateral amygdala, left inferior occipital gyrus, and right middle temporal gyrus during emotional processing, across all paradigms (p < 0.005). The percentage of patients using medication correlated negatively with activation in the right amygdala and left inferior occipital gyrus in patients compared to HC (p < 0.005). Patients with higher symptom severity showed significantly increased activation in the right rostral sgACC, the left medial prefrontal cortex and the right precuneus (p < 0.005). Studies with a higher rate of comorbidity with anxiety and mood disorders also found more pronounced activation in the right putamen, amygdala, and insula as well as decreased activation in the left amygdala and right vmPFC in patients compared with HC (p < 0.005).
*Emotion Regulation*									
	De Wit et al. (130)	Case-control fMRI	OCD = 43 [38.4 ± 10.0; 21M:22F] HC = 38 [39.6 ± 11.4; 18M:20F]	Current or past psychosis was considered as exclusion criteria. 56% of patients met criteria for current comorbid Axis I diagnosis.	Medication-free for at least 4 weeks. Therapy not reported.	Emotion regulation task + ERQ	Fear, neutral, OCD-related	SCID/Y-BOCS/PI	Compared to HC, patients showed higher distress ratings for fear and OCD-related stimuli (p < 0.001). HC and OCD did not differ in fear regulation, but patients had a significantly larger regulation effect on OCD-related stimuli (p < 0.01). In patients, Y-BOCS score correlated with ERQ reappraisal (p=0.001). During emotion provocation patients compared with HC showed an increased amplitude and/or altered timing of the BOLD response in the right amygdala (p_FWE-SVC_=0.004) and occipital cortex at the uncorrected level. During emotion regulation, patients showed decreased activity in left dlPFC (p_FWE-SVC_=0.009) in fear regulation, and increased dmPFC activity (p_FWE-SVC_=0.001) in OCD-related regulation. In patients regulation success did not correlate with brain activity. Disease severity correlated inversely with regulation-related activity in bilateral dmPFC (p_FWE-SVC_=0.002) and thalamus (p_FWE-SVC_=0.04).
	Fernández de la Cruz et al. (131)	Case-control Outpatients	HD = 24 HD + OCD = 19 OCD = 17 HC = 20	Psychosis, bipolar I disorder or substance abuse were considered as exclusion criteria.	Not reported.	DERS	Not applicable.	MINI/DY-BOCS/OCI-R	All three clinical groups obtained higher scores on the DERS compared with HC (p < 0.001). Patients obtained higher scores in the domains ‘nonacceptance of emotional responses', ‘impulse control difficulties', ‘limited access to strategies for regulation' and ‘lack of emotional clarity' compared with HC (p < 0.05). In the entire clinical sample there were significant positive correlations between measures of OCD and DERS (p < 0.001).
	Fink et al. (132)	Case-control	C-OCD = 30 [33.27 ± 11.39; 13M:17F] HC = 30 [32.8 ± 11.9; 13M:17F]	Tic disorder, psychotic or bipolar disorder, and substance abuse were considered as exclusion criteria. Comorbidities: MDD (n=4), MDD with partial remission (n=7), dysthymia (n=1), panic disorder (n=1).	SSRI (n=12), SNRI (n=2), tricyclic antidepressant (n=4). Therapy: outpatient treatment (n=1), inpatient treatment (n=28).	ERQ	Disgust	DSM-IV criteria/Y-BOCS/SCID	Compared to HC, patients scored significantly lower on ERQ subscale cognitive reappraisal (p < 0.001) and significantly higher on expressive suppression (p=0.001).
	Paul et al. (133)	Case-control Outpatients EEG	OCD = 24 [31.7 ± 9.1; 11M:13F] HC = 24 [31.2 ± 8.2; 11M:13F]	Presence of comorbid disorders other than anxiety or Axis II disorders (apart from borderline personality disorder) was considered as exclusion criteria. Comorbidities: agoraphobia with panic disorder (n=1), specific phobia (n=1), social phobia (n=1), adjustment disorder (n=1), OC personality disorder (n=2).	SSRI (n=9). Therapy: CBT (n=9).	ERQ and CERQ + emotion regulation task	Aversive, OCD-related, neutral	Y-BOCS/Y-BOCS Symptom Checklist/OCI-R	Patients scored significantly lower in the CERQ subscale positive refocusing (p < 0.001) and in the ERQ subscale reappraisal (p < 0.001), and higher in the CERQ subscale catastrophizing (p=0.001) than HC. OCD showed a significant LPP enhancement for OCD-related relative to neutral pictures (p=0.003), which was not present in HC. While HC showed significantly reduced LPP amplitudes during both distraction and reappraisal, patients showed a LPP reduction during distraction at trend level (p=0.08), but no significant LPP attenuation during reappraisal (p > 0.99).
	Picò-Pérez et al. (134)	Case-control Resting-state fMRI	OCD = 73 [37.74 ± 10.19; 43M:30F] HC = 42 [39.43 ± 9.79; 22M:20F]	MDD (n=10), GAD (n=3), eating disorder (n=3), tics (n=3), panic disorder (n=2), dysthymia (n=2), OC personality disorder (n=2), ADHD (n=1), agoraphobia (n=1), gambling disorder (n=1).	Fluoxetine (n=13), escitalopram (n=5), sertraline (n=3), fluvoxamine (n=2), paroxetine (n=1), clomipramine (n=8), SSRI + clomipramine (n=12), SSRIs combinations (n=1), antipsychotic augmentations (n=22).	ERQ	Not applicable.	Y-BOCS	OCD scored significantly higher in suppression (p < 0.005) and lower in reappraisal (p < 0.0005) compared to HC. Compared to patients, HC showed higher connectivity between the right amygdala and the right postcentral gyrus (p < 0.05 FWE-cluster corrected). The connectivity between these two regions was significantly correlated with Y-BOCS scores in the patient group (p=0.009). In the OCD group there was a negative association between suppression and functional connectivity between the left amygdala and the precuneus and the bilateral angular gyri (p < 0.05 FEW-cluster corrected).
	Yap et al. (135)	Case-control	OCD = 59 [32.88 ± 10.45; 26M:33F]; HC = 59 [32.81 ± 10.34; 26M:33F]	More than one comorbid condition (n=11), depressive disorders (n=28), AD (n=15), hoarding disorder (n=2), bipolar disorder (n=2), autism (n=2), schizoaffective disorder (n=1), alcohol use disorder (n=1).	Not reported.	DERS	Not applicable.	DOCS/DSM-5 criteria/Y-BOCS	OCD scored significantly higher on DERS total (p < 0.001) and subscales of nonacceptance (p=0.014), goals (p < 0.001), impulse control (p=0.007) and strategies (p < 0.001), compared to HC. Significant group differences were found also for DERS-aware (p=0.004) and DERS-clarity (p < 0.001), but these differences did not remain significant after controlling for depression and anxiety. There were no significant associations between any DERS subscale and Y-BOCS.

OCD, obsessive-compulsive disorder; HC, healthy controls; SSRI, selective serotonin reuptake inhibitor; CBT, cognitive-behavioral therapy; MDD, major depressive disorder; AD, anxiety disorder; DSM, Diagnostic and Statistical Manual of Mental Disorders; PI, Padua Inventory; Y-BOCS, Yale-Brown Obsessive Compulsive Scale; SCID, Structured Clinical Interview for DSM Axis I Disorders; BPRS, Brief Psychiatric Rating Scale; OCI-R, Obsessive-Compulsive Inventory Revised; DASS, Depression Anxiety Stress Scale; DOCS, Dimensional Obsessive-Compulsive Scale; SNRI, serotonin norepinephrine reuptake inhibitor; NAcc, nucleus accumbens; CGI, Clinical Global Impressions severity and improvement scores; MOCI, Maudsley Obsessive-Compulsive Inventory; ADIS-R, Anxiety Disorders Interview Schedule Revised; ERN, error-related negativity; sgACC, subgenual anterior cingulate cortex; vmPFC, ventromedial prefrontal cortex; OFC, orbitofrontal cortex; MINI, Mini International Neuropsychiatric Interview; DY-BOCS, Dimensional Yale-Brown Obsessive Compulsive Scale; DERS, Difficulties in Emotion Regulation Scale; ERQ, Emotion Regulation Questionnaire; dlPFC, dorsolateral prefrontal cortex; dmPFC, dorsomedial prefrontral cortex; C-OCD, contamination-related subtype of OCD; ERC, Emotion Regulation Checklist; CERQ, Cognitive Emotion Regulation Questionnaire; LPP, late positive potential; GAD, generalized anxiety disorder; ADHD, attention deficit hyperactivity disorder; NDRI, norepinephrine-dopamine reuptake inhibitor; PG, pathological gambling; SSNRI, selective serotoninergic and noradrenergic reuptake inhibitors; NaSSA, noradrenergic and specific serotoninergic antidepressants; AN, anorexia nervosa; CD, conduct disorder; PFC, prefrontal cortex; BOLD, blood oxygenation level dependent; SAD, social anxiety disorder; PTSD, post-traumatic stress disorder; Dacc, dorsal anterior cingulate cortex.

### Emotion Experience

There is an abundance of evidence from neuroimaging studies demonstrating that patients with OCD show altered reactivity to emotional stimuli in nonsocial contexts. For example, a recent meta-analysis, including 25 studies with a total of 571 patients and 564 controls, showed that, compared to controls, patients experience increased activation in limbic, frontal, and temporal areas (bilateral amygdala, right putamen, orbitofrontal cortex, ACC, ventromedial prefrontal cortex, middle temporal, and left inferior occipital cortices) during the processing of aversive or symptom-provoking (versus neutral) stimuli (129), indicating heightened emotional reactivity.

Additionally, several studies indicate decreased neural sensitivity to rewarding stimuli, and increased sensitivity to stimuli indicating loss, using gambling (120), risky choice (117), monetary incentive delay (121, 123–125), probabilistic learning (128), or other incentive paradigms (126). For example, studies have shown reduced neural sensitivity in the nucleus accumbens (119, 121) and ACC (125) in response to anticipated rewards, and increased activity in the insula (120, 123) and lateral and medial frontal cortex during anticipated loss (123, 125). Decreased functional connectivity between the nucleus accumbens and limbic areas such as the amygdala during the anticipation of gain and loss has also been observed (124). Additionally, the direct processing of rewarding outcomes has been associated with decreased responsiveness in right medial and lateral orbitofrontal cortex (128) as well as in the caudate nucleus (119, 128). More widespread activation in the frontostriatal circuit including the putamen, precentral cortex, posterior insula, and ACC as well as cerebellum, in response to rewards has been reported as well (123). The processing of positive feedback and monetary reward has also been associated with decreased activation in frontal regions and the posterior cingulate [PCC; (126)]. In addition, the processing of rewards has been related to increased functional connectivity between the left PCC and the right ventromedial prefrontal cortex as well as between left and right PCC (126) and decreased connectivity between frontal and limbic regions (119).

Other studies using probabilistic learning tasks have demonstrated increased prediction error-related activation in the ACC (122, 127) and right putamen (122) during the omission of expected reward, while the unexpected receipt of reward has been associated with increased activity in the nucleus accumbens of patients (127). Studies on performance monitoring in OCD patients have also consistently shown enhanced amplitudes of the ERN during the commission of errors [see (13)], which may also be considered as aversive, negative stimuli or events. This ERP component has been suggested to represent a prediction error signal as it is generated in the ACC and likewise reflects a worse-than-expected outcome [see (136)] that has been found to scale with the emotional significance of the outcome [see (137)]. This suggests that increased ERNs in OCD patients are indicative of an increased affective reactivity to errors.

Despite clear indications for altered emotion experience in OCD in individual contexts, less is known about the emotional reactions of individuals with OCD in response to social emotion-inducing stimuli. Several studies have investigated the experience of basic emotions in patients with OCD as indexed by their facial expressions in response to emotion-inducing video clips of social scenarios (114, 117, 118). Mergl et al. (117) showed that patients with OCD demonstrated significantly slower initial velocity of involuntary laughing movements in response to a humorous movie clip of Mr. Bean. Studies by Bersani et al. (114) and Valeriani et al. (118) showed video clips of social scenarios to patients with OCD to elicit specific emotions (amusement, fear, surprise, anger, sadness, disgust). In both studies, patients with OCD generally displayed fewer concordant and more discordant emotions in response to the clips and also showed less facial mimicry of emotions than healthy controls. These responses were similar to those of patients with schizophrenia (114) and the expression of happiness and disgust was especially poor in those with severe compared to mild-to-moderate OCD symptoms (118). Together, these studies indicate that individuals with OCD show less facial expressivity and less appropriate emotional experiences in response to social scenarios eliciting various basic emotions.

Some other studies have focused on social stimuli inducing more complex emotional responses, specifically the subjective experience and neural processing of guilt and shame, two inherently-social emotions, elicited by depicted scenarios of moral transgressions. In a study by Basile, Mancini, Macaluso, Caltagirone, and Bozzali (113), patients with OCD reported to experience more guilt than controls while processing guilt-inducing sentences, especially for sentences indicating guilt derived from transgressing an inner moral rule (deontological guilt) compared to altruistic guilt, which is defined as guilt of having disregarded a personal altruistic goal. The experience of guilt versus nonmoral, basic emotions (anger and sadness) was accompanied by reduced activation in the ACC extending to superior/medial frontal gyrus. According to the authors, the increased rather than decreased activity in this region previously associated with the experience of guilt could be explained by cerebral efficiency, as feelings of guilt are more frequently experienced in patients with OCD. In a comparable task, patients with OCD showed higher activation than controls in various regions including the superior frontal- and precentral gyrus, cingulate gyrus, superior temporal gyrus and decreased activation in anterior cingulate while processing guilt-inducing compared to neutral sentences (116). Symptom severity (Y-BOCS) was positively associated with activation of left middle frontal gyrus and temporo-parietal junction during the experience of guilt. Shame on the other hand was associated with increased activation in the uncus, parahippocampal gyrus, and middle temporal gyrus, as well as the hypothalamus, and decreased activity in the middle frontal gyrus and inferior parietal lobe in patients compared to controls. Thus, the authors showed that the experience of shame and guilt was associated with increased reactivity in a widespread neural network. On the behavioral level, patients did not report to experience more guilt and shame in the experimental task, although self-report questionnaires did demonstrate generally higher levels of guilt and shame in patients, which the authors suggest may indicate an increased sensitivity to social norms. Fontenelle et al. (115) used multivariate pattern analysis to identify brain regions that discriminate OCD patients from controls across different moral emotions evoked while reading different scripts. They showed that several brain regions including the nucleus accumbens, lingual gyrus, and middle temporal gyrus, were able to discriminate patients from controls across distinct moral emotions (guilt, compassion, anger, and disgust). Together, these studies suggest that patients with OCD tend to experience more guilt in response to (moral) emotion-evoking stimuli (113), and show altered neural processing of such stimuli (113, 115, 116).

### Emotion Regulation

Several studies have investigated emotion regulation skills in OCD, all of which are limited to nonsocial contexts. These studies have largely focused on self-report or observer-reported measures, such as the Emotion Regulation Questionnaire [ERQ; (138)]. The ERQ focuses specifically on cognitive reappraisal, which refers to the tendency to change the interpretation of an emotion-eliciting situation so that it diminishes its negative impact, and expressive suppression, which refers to a more maladaptive emotion regulation strategy that consists of the inhibition of emotion-expressive behavior. Fink, Pflugradt, Stierle, and Exner (132) and Picó-Pérez et al. (134) showed that OCD patients make less use of reappraisal and more use of suppression techniques. Picó-Pérez and colleagues additionally demonstrated using resting-state functional connectivity analyses with the left and right amygdala as seed regions, that within patients, suppression was negatively related to connectivity between the left amygdala, the precuneus and the bilateral angular gyri. These findings thus suggest that impaired parietolimbic connectivity may be associated with the preferential use of maladaptive emotion regulation techniques.

Other studies likewise demonstrated self-reported emotion regulation impairments in OCD patients using the Difficulties in Emotion Regulation Scale (DERS), a questionnaire that focuses not only on the modulation of emotions but also more generally on the awareness, understanding, and acceptance of emotions (139). The DERS consist of six subscales: (1) nonacceptance of emotional responses; (2) difficulty engaging in goal-directed behavior when distressed; (3) impulse control difficulties when distressed; (4) lack of awareness of emotions; (5) limited access to (adaptive) strategies for regulation; and (6) lack of emotional clarity. Fernández de la Cruz et al. (131) showed that patients compared to controls had significantly higher scores on all subscales except for the “lack of emotional awareness scale.” Similarly, Yap et al. (135) found that OCD patients scored significantly higher than controls on all DERS subscales, and group differences remained significant after correcting for depression and anxiety on all scales except for the lack of emotional awareness and emotional clarity scales. These findings indicate that patients with OCD have difficulties regulating their emotions, specifically expressed in the tendency to show a nonacceptance of emotions, experienced difficulties in goal-directed behavior and impulse control when distressed, and the use of maladaptive regulation strategies. Additionally, these difficulties seem at least partly independent of more general depressive or anxious symptoms.

Two studies employed emotion-provocation paradigms to assess the neural correlates of emotion regulation in patients, and indicate that patients show altered neural activity during emotion regulation (130, 133). In an fMRI study by De Wit et al. (130), patients and controls viewed general- and disorder-specific emotion-provoking stimuli, and were instructed to either attend these stimuli or to regulate their emotions through cognitive reappraisal. OCD patients gave higher ratings of distress after viewing emotion-provoking stimuli, which was accompanied by amygdala-hyper responsiveness, but comparable distress reduction as control after instructed emotion regulation. During emotion regulation, OCD patients showed diminished left dorsolateral prefrontal cortex activity and increased left dorsomedial prefrontal activity compared to controls, which may indicate the use of alternative or compensatory emotion regulation mechanisms. They also showed less frontal-amygdala connectivity than controls, which the authors proposed may be reflective of a generally diminished ability to effectively regulate pathological anxiety. Using a similar task, Paul et al. (133) assessed the electrophysiological correlates of emotion regulation. Compared to controls, OCD patients had higher arousal ratings after viewing symptom-provoking stimuli as well as enhanced amplitudes of an event-related potential called the late positive potential (LPP) while viewing these images. The LPP is thought to reflect facilitated attention to emotional stimuli, and has been found to be modulated by emotion regulation strategies (140). Indeed, healthy controls showed reduced LPP amplitudes after instructed emotion regulation. However, patients with OCD did not show a reduction in the LPP during cognitive reappraisal, despite the fact that subjective arousal ratings were successfully reduced. Self-reported emotion regulation skills were also assessed, using the ERQ and the Cognitive Emotion Regulation Questionnaire [CERQ; (141)]. The CERQ focusses on cognitive (i.e., explicit) emotion regulation strategies, and consists of nine different scales, of which four focus on more maladaptive or dysfunctional strategies (self-blame, focusing on thought/rumination, catastrophizing, blaming others), and of which five are thought to represent somewhat more adaptive methods (acceptance, positive refocusing, refocus on planning, positive reappraisal, putting in perspective). Here too, patients indicated poorer self-reported emotion regulation skills as indicated by lower scores on the reappraisal subscale of the ERQ as well as lower scores on the positive refocusing subscale and higher scores on the catastrophizing subscale of the CERQ.

### Section Summary and Discussion: Emotion Experience and Regulation

Research clearly indicates that the experience of emotions in patients with OCD is altered. Patients with OCD show heightened affective reactivity and altered neural processing of various emotion-inducing and emotion-provoking stimuli, show decreased neural sensitivity to reward and heightened (prediction) error responses. Less is known about emotion experience in social contexts. Some studies indicate that patients show less appropriate emotional experiences and facial expressivity in response to emotion-inducing social scenarios. These studies may for example suggest that patients with OCD experience less emotional contagion, which is the automatic mimicking and synchronizing of facial expressions, vocalizations, postures, and movements with others leading to similar emotions (100). Alternatively, it has also been put forward that these incongruent responses could reflect an increased effort to suppress or resist unpleasant emotions (142) and may therefore reflect emotion regulation attempts. Yet, still alternative explanations are possible. The use of medication such as antidepressants has for example been associated with alterations in emotion experience, such as emotional blunting (143). The impact of different kinds of medications should therefore be explored further. Nevertheless, these studies suggest that observable basic emotional responses to various social situations are disturbed in OCD. Additionally, studies have indicated that patients experience increased levels of more complex social emotions such as guilt and altered neural processing of various moral emotions compared to healthy controls, which seems in line with theories of OCD that highlight the role of responsibility, guilt and shame in the etiology of the disorder. For example, the cognitive theory of OCD suggests that patients misinterpret intrusive thoughts as indicating that they are responsible for preventing harm coming to others or oneself, which in turn triggers actions such as compulsions to prevent feared events (144). Similarly, it has been argued that patients are characterized by a fear of guilt resulting from behaving irresponsibly and/or from not behaving responsibly, which in turn triggers compulsive symptoms (145).

Many studies additionally show that OCD patients employ more maladaptive emotion regulation skills, and that these effects seem largely independent of comorbid depression and anxiety levels. There is also evidence for altered neural activity during emotion regulation in patients (130, 133), which may point to the use of compensatory or (inefficient) alternative emotion regulation strategies.

To conclude, studies indicate that patients with OCD are characterized by increased emotional reactivity and poor emotion regulation abilities. These emotional disturbances may be triggered by external factors or stimuli, such as in the studies discussed. However, patients with OCD often also experience emotions that are not specifically triggered by the social context but which are rather elicited by more internal processes such as obsessive thoughts. If patients are unable to effectively regulate these emotions, this will unequivocally impact how individuals with OCD interact with their environment. Yet, currently, research on the experience and regulation of emotions in various social contexts is still lacking.

## Discussion

**Figure 1:**
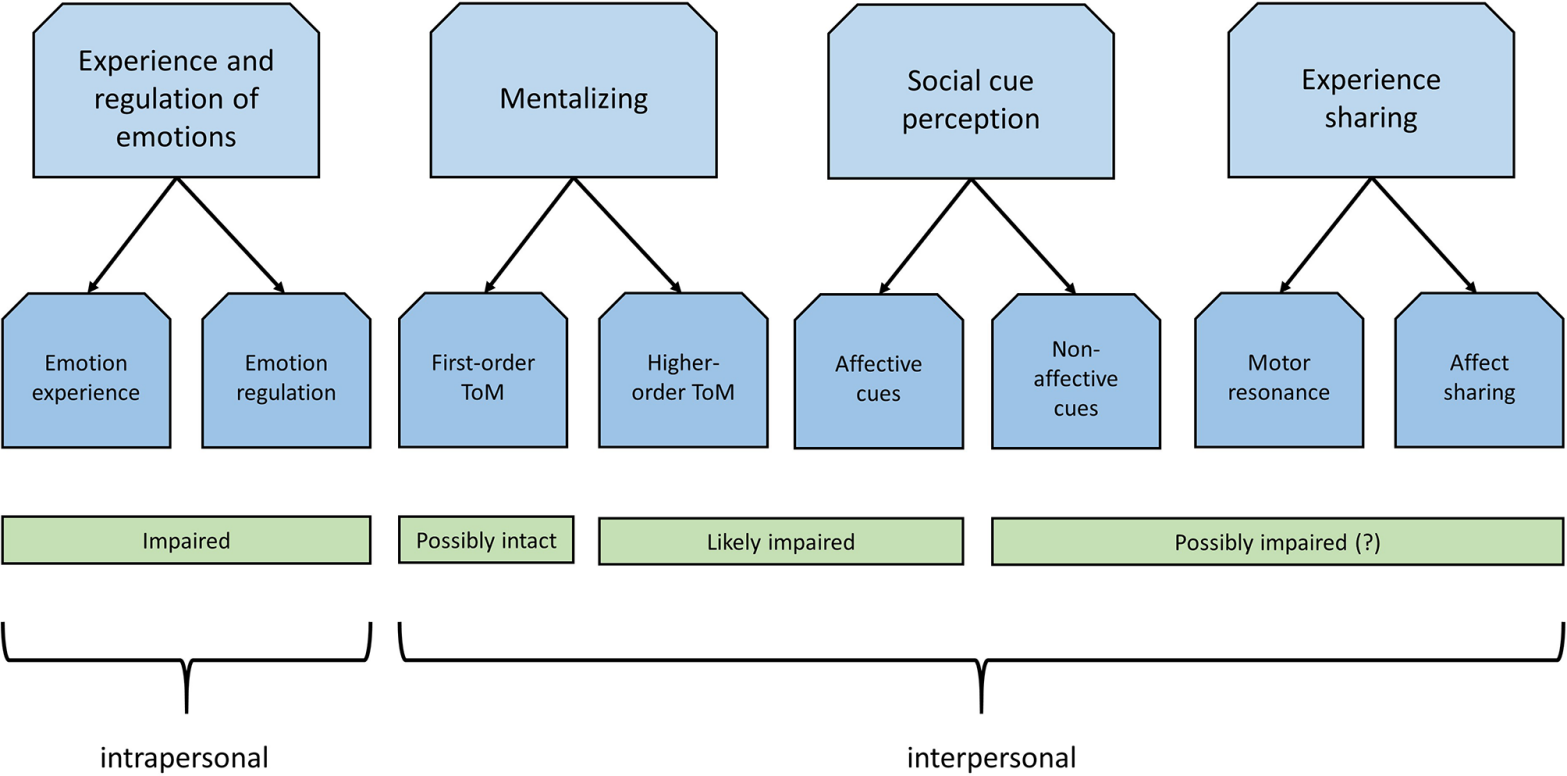
Schematic overview of social cognitive alterations in obsessive-compulsive disorder with regard to the domains discussed in the current review, based on Green et al. (4). These domains can be divided in intrapersonal and interpersonal domains. Much research has been conducted on the intrapersonal domain, providing strong evidence from neuroimaging studies for altered emotion experience and impaired emotion regulation. Evidence with regard to the interpersonal domain however is limited and less consistent. Affective cue perception is likely impaired, specifically with regard to the recognition of facial expressions of disgust. Similarly, there is evidence for theory-of-mind (ToM) impairments especially with regard to higher-order inferences. There is also some evidence for impaired perception of nonaffective cues, although research is scarce. Studies on experience sharing are lacking, though there are some indirect indications that these domains may be affected as well.

In the current review, we aimed to offer an overview of the relation between social cognition in patients with OCD. Overall, these studies indicate that patients are characterized by social cognitive alterations in almost all domains suggested by Green et al. (4). Evidence indicates that OCD patients show deficits in the perception of social cues, specifically with regard to the recognition of facial expressions of disgust, and also show altered neural processing of facial emotions. There are also indications that patients are characterized by deficits in nonaffective social cues, such as deficits in the recognition and perception of nonaffective social cues, such as biological motion and body poses implying action in OCD patients. However, studies in this domain are scarce and may be subjected to publication bias. Furthermore, there is support for deficient ToM or mentalizing abilities in patients with OCD, which may be particularly pronounced in those with poor illness insight. Studies on motor resonance and affect sharing OCD are lacking. Impaired imitation of other's actions has been reported, which, together with observed deficits in the perception of biological motion or action, may point to deficient motor resonance and impaired functioning of the MNS, yet this remains to be investigated. Additionally, self-report studies indicate that patients with OCD experience increased empathic distress when confronted with the distress of others, or similar emotional congruence, suggesting that affect sharing is intact, and possibly exaggerated. On a more intrapersonal level, there is convincing evidence that patients with OCD show heightened affective and altered neural reactivity to emotional stimuli, and have poor emotion regulation skills, which may also have important repercussions for social interactions. Following the example of Green et al. (4), **Figure 1** provides a schematic overview of the social cognitive disturbances in OCD as discussed in this review. A word of caution is necessary however, as findings are inconsistent and many social cognitive domains remain underexplored, which makes it difficult to draw firm conclusions with regard to a social cognitive profile associated with obsessive-compulsive symptomatology. It should also be noted here that the current review addressed only a limited range of domains relevant for daily-life social functioning, and there may be many more processes relevant to OCD that could affect social functioning. However, in this review we decided to focus specifically on the domains as demarcated by Green et al. (4).

Nevertheless, the social cognitive deficits that were found may in part explain why patients with OCD experience such poor quality of life on social domains. Problems in the ability to recognize basic social cues such as facial expressions and biological motion and the ability to understand more advanced mental states carry obvious implications for social functioning, as these abilities are critical in order to navigate the social environment in an adaptive manner. The current review additionally demonstrated that on a more intrapersonal level, patients with OCD are characterized by heightened emotional and neural reactivity as well as by problems in emotion regulation, which may directly contribute to the development and maintenance of obsessions and compulsions [see, e.g., (111)], but which may also importantly hinder the enjoyment of social relations and contribute to maladaptive social behavior. For example, the elevated scores on empathic personal distress indicate that patients with OCD also display a heightened emotional reactivity to social stimuli and situations. Indeed, emotion regulation is critical in order to show adaptive empathic or prosocial reactions to experiences of others and is thus considered a critical component of relationship formation and maintenance. Given that emotions are often regulated with the goal of influencing social situations and interaction partners within a social context (146), regulation of emotions in a social context is arguably much more complex than when one does not have to deal with this context. Until now, however, the experience and regulation of emotions has mainly been investigated in nonsocial contexts. This is surprising as the social context can be an important source of emotions. This seems particularly true for individuals with OCD, who experience difficulties in managing their daily lives due to the invalidating and time-consuming nature of their symptoms. This can also put a huge strain or burden on family members and loved ones, who sometimes engage in symptom accommodation in order to help patients with their anxiety and/or in order to avoid conflict, which may in turn contribute to worsening of symptoms [e.g., (147)]. Perceived or experienced stigmatization may likewise represent an important social trigger of feelings of shame or embarrassment (148). Additionally, there is evidence indicating that the obsessions and compulsions from which patients with OCD suffer often have a social component in itself. Patients with OCD often show increased feelings of responsibility or guilt for how their actions may affect others (149). They might for example have intrusions about hurting someone they love, resulting in feelings of guilt and avoidance of this loved one to prevent harm. Moreover, patients might be afraid that something bad will happen to a loved one if a certain ritual is not carried out, even though they recognize the irrationality of this behavior. The cognitive theory of OCD highlights this inflated sense of responsibility for other's harm as it suggests that patients misinterpret intrusive thoughts as indicating that they are responsible for preventing harm coming to others or oneself, which in turn triggers actions such as compulsions to prevent feared events (144). Importantly, these symptoms are thought to form an important obstacle for enjoyable and successful social interactions. Moreover, anxiety or distress triggered by symptoms itself rather than the social context, can also impact how patients deal with their social environment if patients are unable to effectively regulate these emotions. Extending investigations on the symptomatology of OCD from an individual to a social context is therefore highly important for future investigations as it may importantly contribute to our understanding of the symptomatology and social difficulties in daily life of patients.

The fact that several individual studies do not indicate any social cognitive deficits, such as facial emotion recognition and ToM impairments, suggests that these deficits may only be present or are more pronounced in a specific subset of patients, although in some cases statistical power may also play a role. An important target for future studies is therefore to unravel which characteristics of patients are associated with poorer social cognitive functioning. A promising factor in this respect is level of illness insight of patients, as several studies show deficient ToM abilities only in those with less insight (73, 75, 80). However, the role of factors related to poor insight, such as increased comorbidity with schizophrenia, or poorer overall cognitive, emotional or intellectual functioning, needs to be investigated as well. Deficits in more general cognitive abilities often found in patients with OCD may also contribute to social cognitive difficulties. For example, cognitive skills such as reasoning and problem solving are thought to be necessary in order to make accurate ToM inferences, and impairments herein may thus also affect social cognitive processes (14). It is therefore possible that ToM impairments in OCD patients are primarily an indirect result of more prominent deficits in general cognitive abilities. Relatedly, medication or treatment status may also help explain incongruent findings. Studies by Lochner et al. (45) and Rector et al. (49) indicate that medication or psychological treatment might affect (i.e. improve) the ability to recognize facial expressions of disgust, as these studies showed higher recognition scores after SSRI and CBT treatment. Yet, many studies including OCD patients did not report whether they were receiving any concurrent medications or treatment, and it is currently unknown how different types of medication may impact emotion recognition. The use of medication could also affect other social cognitive processes, as antidepressants are for example known to have an effect on more general cognitive functioning, such as attention, executive functioning and memory (150). These results stress the importance of taking treatment status into account when assessing emotion recognition as well as social cognitive skills in general. Lastly, given that OCD is a heterogeneous disorder with many different manifestations, different subtypes may be associated with different social cognitive profiles. Yet, current investigations of subdimensions have been rather inconclusive. This may be explained by the fact that these studies have been largely limited to small samples and a focus on overt symptoms (e.g., checking or cleaning) of the disorder rather than on underlying reasons for these behaviors. Importantly, underlying motivational dimensions such as “harm avoidance” and “incompleteness” may be a more fruitful approach to clarify heterogeneous findings in OCD (151). Whereas “harm avoidance” seems to represent a more anxiety-focused motivation to prevent harm, “incompleteness” refers to a more sensory-affective motivation where individuals feel that actions are incompletely achieved that are more closely related to perfectionism and obsessive-compulsive spectrum disorders. Such motivational and orthogonal dimensions of OCD might represent a more valuable approach to explain social cognitive heterogeneity than more categorical, behaviorally driven subtype characterizations. In summary, important moderating factors that might help unravel heterogeneity in findings include level of illness insight, comorbidities (e.g., schizophrenia, depression), nonsocial neurocognitive functioning, medication or treatment status, and symptom dimensions.

Besides characteristics related to patients, characteristics of the tasks may also contribute to the inconsistencies in results. A wide variety of different tasks have been used to assess the same social cognitive domain, which makes comparison across studies difficult. For example, emotion recognition tasks differed with regard to the nature of the expressions (e.g., static versus morphed), the stimuli set, and the specific task instructions (e.g., labelling versus matching). The number of trials presented also varied considerably. For example, Kornreich et al. (43) presented only 12 trials with facial expressions whereas Jhung et al. (42) and Kang et al. (103) presented as much as 360 trials. Factors like this not only limit the comparability of results between studies but also raise questions with regard to the validity and reliability of the tasks employed. More standardized test batteries are needed to draw out a clear social cognitive profile across the various subdomains of social cognition, which will allow for better comparisons across studies and disorders.

While it has been shown that several social cognitive tasks, especially assessments of ToM, have high test-retest or interrater reliability [see, e.g., (152)], the extent to which impairments on the various social cognitive tasks that OCD patients exhibit are valid indications of social cognitive problems in daily life is currently unclear. Notably, effect sizes for disgust recognition deficits in OCD patients were much smaller for tasks employing morphed compared to static facial expressions (41), whereas the first can be seen as the most ecologically valid and subtle assessment of emotion recognition. Furthermore, many tasks focus on a specific aspect of social cognition (e.g., the ability to identify emotions from either facial expression or vocal or narrative information), whereas in real life individuals need to integrate all these different modalities (e.g., facial, bodily, paralinguistic, auditory and contextual cues) to make sense of others and to function in a socially appropriate way. Only one study used such a multimodal task in OCD patients (74). Interestingly, this study showed no differences in performance between patients and healthy controls. On the one hand, the integration of different processes or modalities may result in higher complexity and cognitive load, such as during higher-order ToM inferences. On the other hand, it possible that the availability of cues from multiple modalities helps compensate for deficits in specific modalities, due to an increased richness of the environment. There are several other multimodal tasks available [see, e.g., (152)], which could help assess social cognitive functioning in a more ecologically valid manner.

The extent to which observed social cognitive deficits are specific to OCD or can be seen as more transdiagnostic deficits that contribute to psychopathology in general should also be investigated in more detail. For example, a recent meta-analysis of 30 different clinical disorders demonstrated social cognitive deficits across practically all these disorders (153). A more standardized test battery covering multiple social cognitive domains may help more clearly elucidate differences and communalities across disorders. The observed bias of OCD patients to assign more negative valence to faces may well be related to comorbid mood disturbances as this is something also commonly found in depression (64). Likewise, problems with mentalizing and altered emotion experience and regulation have been reported in many other disorders as well (153, 154). Importantly, only a subset of the reviewed studies included comorbid diagnoses or symptoms as covariate in their analysis or considered the presence of comorbidities as an exclusion criterion (see **Tables 1**–**3**). On the other hand, some of these deficits, such as problems in emotion regulation, were found to remain after taking comorbid symptoms such as depression and anxiety into account, suggesting that they form a unique part of the symptomatology of the disorder. In addition, specific deficits in the recognition of disgusted faces and a bias to perceive ambiguous faces as expressing disgust, for example, have not been reported in other disorders, and thus seem to represent a rather unique aspect of obsessive-compulsive symptomatology.

Findings from the current review may have important clinical implications as the identified social cognitive deficits represent important targets for intervention. There are for example facial emotion recognition trainings available (155) which may help remediate disgust recognition deficits in patients. Similarly, trainings exist with regard to ToM (156, 157) and emotion regulation [e.g., (158)], and there is evidence that compassion training may help overcoming empathic personal distress (159). Whether such interventions may also effectively reduce symptomatology and daily life problems in social functioning in OCD remains to be investigated. Tackling social (cognitive) problems in OCD is of critical importance, as poor social functioning has been associated with, among other things, poorer quality of life, and poorer functional outcomes including more severe symptoms, and a higher number of psychiatric comorbidity (10). The social aspects and impact of OCD are therefore not something to be ignored.

## Toward a Social Neurocognitive Interactive Account of OCD

**Figure 2:**
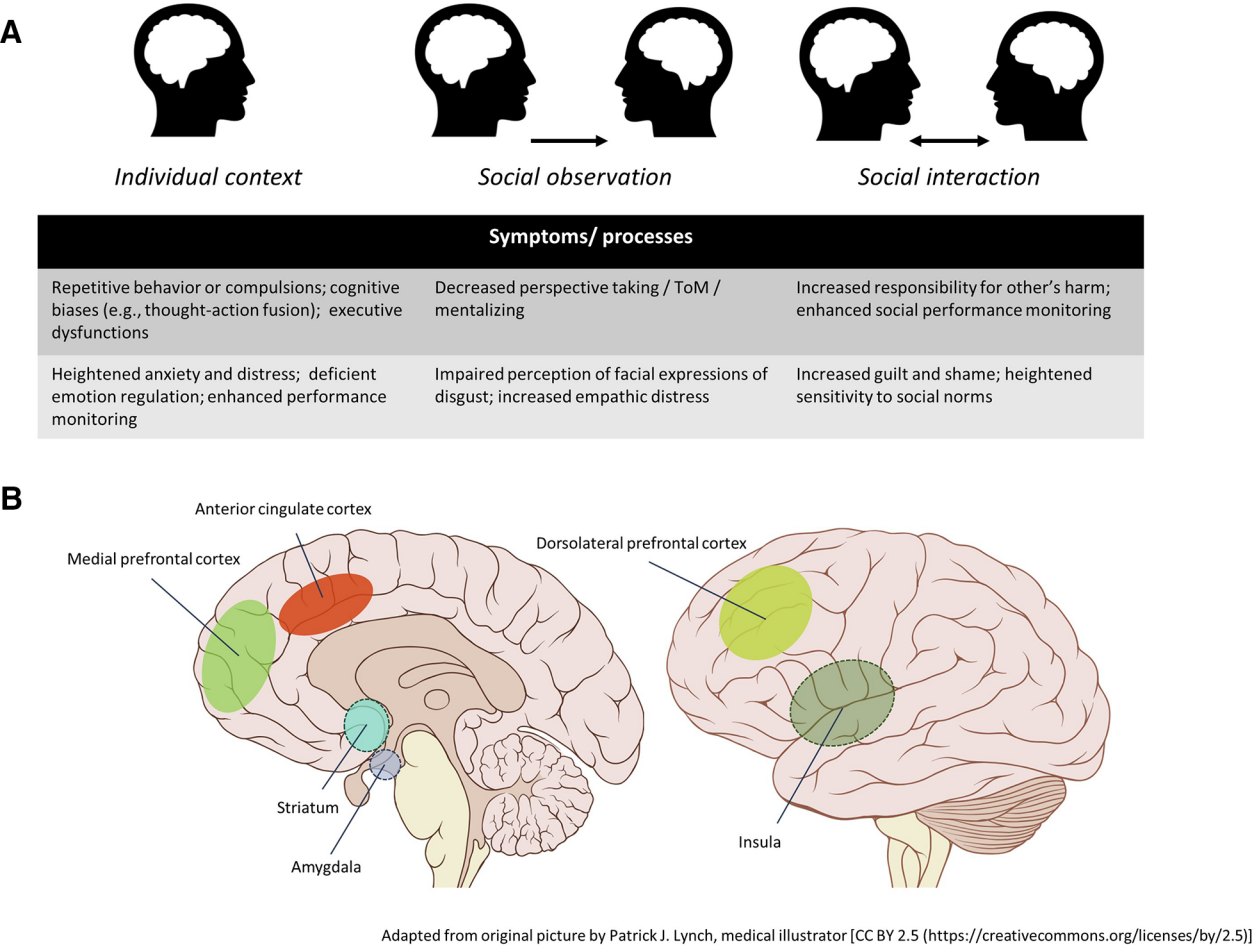
Overview of symptoms and processes of obsessive-compulsive disorder discussed in this review **(A)**, from an individual context *via* a social observational and finally toward an interactive context, as well as hypothesized brain regions primarily implicated in these social alterations based on the current review **(B)**. Increased activity in the anterior cingulate cortex and insula in patients has been reported during the processing of the negative or aversive stimuli, as well as during negative prediction errors and error processing more generally. Reduced responsiveness of the striatum (specifically nucleus accumbens) has been demonstrated during the processing of positive prediction errors and rewards. Altered amygdala activity has been reported during the processing of emotional stimuli and fearful or threatening facial expressions. Reduced activation of the dorsolateral prefrontal cortex has been observed during emotion regulation. Although studies on the neural correlates of mentalizing/theory of mind (ToM) are lacking, studies in healthy volunteers have consistently implicated the medial prefrontal cortex in this process. Given that deficits in ToM have been observed in obsessive-compulsive disorder, this region may also be affected in the disorder.

Available measures of social cognition have been criticized as they are limited to a “spectator” account of social cognition, whereby individuals merely observe others while thinking about their mental states, instead of interacting with them (1, 160). Schilbach et al. (160) argue that social interactions importantly contribute to our understanding of the mental states of others and that social cognition might be fundamentally different when we are in active interaction with others compared to when we are solely observing others. In social interaction, we might depend on more implicit, automatic, and spontaneous emotional processes rather than explicit cognitive inferences to understand others and there is evidence for a dissociation between such implicit and explicit levels of social cognition (1, 161). Patients with high-functioning autism, for example, generally show reduced implicit or spontaneous inferences of others mental states, despite showing intact explicit cognitive mental attributions [e.g., (162, 163)], suggesting that they are mainly characterized by a problem of social interaction (1). This seems relevant to patients with OCD as well. More often than during observation, social interactions involve an emotional component, and in an interactive context it is essential to regulate these emotions in such a way that relations with others are facilitated. Given that patients with OCD show heightened affective reactivity and social emotions such as inflated feelings of responsibility and guilt, as well as poor emotion regulation skills, this may be particularly challenging for patients with OCD. Moreover, during social interaction, many different cognitive processes need to be integrated in an ongoing fashion in order to behave in an adaptive manner, as one does not only need to take own actions, thoughts and emotions into account, but also the actions, thoughts and emotions of others, as well as their effect on the self, and vice versa. To get a better perspective on daily-life disturbances in OCD, it is therefore important to not only study social cognition in these patients from an observer's perspective, but to additionally start focusing on more implicit and interactive paradigms (see **Figure 2A** for a schematic overview).

Neuroimaging methods may aid the investigation of more implicit and interactive social cognitive processes, as such methods do not require explicit prompting or responding. For example, recent advances in the field of virtual reality provide exciting new opportunities for mimicking realistic social interactions in the MRI scanner [see, e.g., (164)]. However, although recent studies have started using neuroimaging techniques to investigate social cognition in OCD, most studies so far have focused on behavioral assessments. Future studies using neuroimaging techniques are needed to gain more insight into the neural mechanisms underlying altered social cognitive processes. Results from the current review demonstrate that patients with OCD show altered neural activity in- and connectivity between brain regions associated with the recognition, experience, and regulation of emotions, such as the amygdala, insula, nucleus accumbens, ACC, and dorsolateral prefrontal areas (see also **Figure 2B**). Importantly, these results show that those brain areas known to be affected in OCD during nonsocial cognitive and affective processes, also seem to be affected during social variants of these processes. Yet, to date, neuroimaging studies on OCD have mainly been limited to nonsocial cognitive processes, while incorporating the social context in cognitive neuropsychiatric investigations may importantly advance our understanding of the social and functional impairments that characterize OCD patients. A promising candidate in this respect is performance monitoring. As mentioned in the introduction, research has consistently shown enhanced ERN amplitudes in OCD. This has led to the suggestion that this enhancement reflects a possible biomarker of the disorder [see e.g., (165)]. However, increased amplitudes of the ERN are not limited to OCD, but are also found in other anxiety disorders as well as in depression [see (13)]. Importantly, with the integration of social context in performance monitoring research, a more disorder- or symptom-specific marker of OCD may be identified. For instance, the heightened feelings of responsibility for harm and interpersonal guilt that characterize patients suggests that patients with OCD might show specifically enhanced monitoring of their own performance in interactive social responsibility contexts, i.e., when their actions directly have consequences for someone else (166). Such enhancements might not be expected for other disorders with more self-focused symptoms such as health anxiety. So-called social performance monitoring paradigms [see e.g., (166–168)] therefore represent a relevant example of an interactive and implicit measure of social cognition that may substantially inform us on possible alterations in social interactive behavior in patients with OCD.

## Conclusion

To conclude, the reviewed studies indicate that OCD seems to be associated with alterations in social cue perception, specifically impaired recognition of facial expressions of disgust and biological motion and actions, poorer mentalizing or ToM skills, possibly suboptimal motor resonance, heighted or altered affective and neural responding, and poorer emotion regulation abilities, all of which are processes that may contribute to deficient social functioning in patients with OCD. This review provides an important first step to drawing out a unique social cognitive profile of OCD. However, findings are somewhat inconsistent, and the number of studies in the various subdomains of social cognition are scarce and difficult to compare due to heterogeneity in participant and task characteristics. Future studies should aim to further explore the role of social cognition in OCD using multimodal and ecologically valid paradigms, with a focus on potential moderating factors and developmental pathways. Finally, investigating social interactive behavior in OCD from a cognitive neuropsychiatric perspective remains an essential endeavor as it may importantly advance our understanding of the symptomatology and daily-life disturbances in this intricate and burdensome disorder.

## Author Contributions

MJ wrote the first version of the manuscript. SO and ED provided feedback and revised the manuscript. All authors approved of the final version.

## Funding

This work was supported by a personal grant from the Netherlands Organization for Scientific Research to ED (NWO; VIDI Grant No. 452-12-005).

## Conflict of Interest

The authors declare that the research was conducted in the absence of any commercial or financial relationships that could be construed as a potential conflict of interest.
